# The Effect of Nb, Ta, and Ti on the Oxidation of a New Polycrystalline Ni-Based Superalloy

**DOI:** 10.1007/s11085-023-10218-7

**Published:** 2024-01-30

**Authors:** J. W. X. Wo, M. C. Hardy, H. J. Stone

**Affiliations:** 1 Department of Materials Science and Metallurgy, University of Cambridge, 27 Charles Babbage Road, Cambridge, CB3 0FS UK; 2 Rolls-Royce plc, PO Box 31, Derby, DE24 8BJ UK

**Keywords:** Ni-based superalloys, Oxidation, Niobium, Tantalum, Titanium

## Abstract

The effect of variations in Nb, Ta, and Ti concentrations in exchange for Al on the oxidation resistance of a new polycrystalline Ni-based superalloy (C19) was studied in air at 800 °C for up to 1000 h. An external scale of Ti-doped Cr_2_O_3_ and a sub-scale of discontinuous Al_2_O_3_ intrusions formed on the surface of all the studied alloys. Contrary to other reports, increasing the Nb concentration improved the oxidation performance and may have promoted the formation of a CrTaO_4_ layer, thereby reducing oxygen ingress. The addition of Ta also significantly improved oxidation resistance and reduced the depth of the Al_2_O_3_ intrusions. Increasing the Ti concentration did not significantly affect the oxidation performance, potentially due to the relatively low Ti concentrations investigated. Several of the studied alloys with modified Ta and Ti concentrations showed regions of continuous Al_2_O_3_ scale formation, suggesting that the compositions are in a transition regime between Cr_2_O_3_-forming and Al_2_O_3_-forming behaviour. The findings suggested that part of the Ti content in C19 could potentially be replaced with Nb, Ta and/or other elements to further enhance oxidation resistance and other desirable properties. Overall, the insights gained could serve as a guide to optimise the composition of C19 and similar alloys for enhanced oxidation resistance.

**Supplementary Information:**

The online version contains supplementary material available at 10.1007/s11085-023-10218-7.

## Introduction

Worldwide efforts to minimise CO_2_ emissions from the aviation industry would benefit from the advent of aeroengines with higher operating temperatures for improved fuel efficiencies. To withstand the harsher oxidation conditions expected at higher temperatures, Ni-based superalloys with superior oxidation resistance are required. Traditionally, Al and Cr are used in Ni-based superalloys to form continuous scales of Al_2_O_3_ and Cr_2_O_3_ for oxidation resistance, respectively [1–3]. Giggins and Pettit [4] showed that alloys based on the Ni–Cr–Al system could be divided into three groups with distinct oxidation behaviours: Type I alloys form a non-protective external scale of NiO with a discontinuous sub-scale of Al_2_O_3_ and Cr_2_O_3_ intrusions; Type II alloys form a continuous external scale of Cr_2_O_3_ with sub-scale discontinuous Al_2_O_3_ intrusions; and Type III alloys form a continuous external scale of Al_2_O_3_. It was also noted [4] that alloys that form a continuous scale of Al_2_O_3_ underneath an external Cr_2_O_3_ scale can also be classified as Type III. While balancing the concentrations of Al and Cr to form continuous Al_2_O_3_ or Cr_2_O_3_ scales remains a core objective of alloy design, it is also necessary to consider the roles of the other alloying elements that could have major influences on environmental resistance. Barrett et al. [5] conducted a comprehensive study on the role of common alloying additions in Ni-based superalloys on oxidation resistance. By assigning an “attack parameter” based on oxidation mass change to a series of alloys with varying compositions, the authors identified several key elemental trends that have become highly influential in oxidation-resistant alloy design. In that study, a concentration of Al ranging from 5 to 8 at.% had the largest beneficial effect on oxidation resistance relative to any other element. In addition, a moderate amount of Cr (10 at.%) and low concentrations of Ti (2 at.%) also resulted in optimal oxidation performance. However, their results suggested that Mo, W, and Ta had mixed effects on the oxidation resistance and required further investigation. A more recent study by Smialek et al. [6] extended the work of Barrett et al. [5] with further examination of the role of Ti, Nb, W, Mo, Co, and Al on oxidation resistance. In their study, it was found that Ti and Nb had the most detrimental effects, while Ta showed a transition from detrimental to beneficial effects with increasing temperature. Therefore, the effects of these elements must be carefully considered when developing new alloys for enhanced oxidation performance.

The effect of Nb on the oxidation behaviour of Ni-based superalloys has been investigated by many authors with mixed results. In one study [7], the addition of Nb to an alloy was shown to increase the oxidation weight gain compared to a Nb-free alloy. However, it was also shown that Nb resulted in a more continuous and compact oxide scale of TiO_2_ and Cr_2_O_3_ that inhibited vaporization and oxide spallation. The results suggested that the addition of Nb presented a trade-off where the resultant oxide scales of TiO_2_ and Cr_2_O_3_ were stabilised in exchange for an overall larger mass gain. The same authors investigated the effect of Nb on the internal oxidation behaviour in another study [8] and showed that the depth of discontinuous Al_2_O_3_ intrusions increased with Nb concentration, therefore expanding the *γ*′-depleted zone. The adverse effects of Nb on oxidation were also corroborated by the work of Alkmin et al. [9]. By substituting Ta for Nb in the Ni-based superalloy MAR-M246, the authors found the Nb-containing alloy to have enhanced oxidation resistance at 800 °C but significantly worse internal oxidation/nitridation effects at 900–1000 °C due to excessive spallation. Reports of an apparent benefit to oxidation resistance with Nb additions have been made in two studies by Weng et al. [10, 11] where an optimal concentration of Nb for maximum oxidation resistance was suggested. In general, these results contradict other sources in the literature that report Nb to have deleterious effects on oxidation resistance [12, 13].

The effect of Ta, in moderate concentrations, has been shown to generally have a beneficial effect on oxidation behaviour in Ni-based superalloys. An early study by Yang [14] showed the addition of Ta to potentially aid the formation of an Al_2_O_3_ scale, but no detailed description of the exact mechanism was provided. In this regard, other researchers [15, 16] have proposed that Ta restricts the outward diffusion of Al and consequently reduces the oxide scale growth. Park et al. [17] investigated the effects of both Al and Ta on the oxidation of Ni-based superalloys at 850 °C and 1000 °C and found mixed results. At 850 °C, improved oxidation resistance was associated with increased concentrations of Al, but the formation of Al_2_O_3_ was hindered by the presence of Ta at 850 °C. However, increased Ta concentrations were shown to be effective at reducing the oxidation mass gains at 1000 °C with seemingly little effect on Al_2_O_3_ formation, suggesting that the higher diffusion kinetics of Al expected at higher temperatures overcame the hindering effect. In another study [18], it was shown that Ta in two commercial Ni-based superalloys combined with Ti during oxidation to form TiTaO_4_, which was associated with a subsequent decrease in Ti diffusion to the alloy-oxide interface.

The detrimental effects of Ti on the oxidation behaviour of Ni-based superalloys are well-known and widely reported [2, 5, 14, 19, 20]. Yang [14] found that a Ti concentration of 1 at.% did not significantly affect the oxidation rate of a complex Ni-based superalloy but a concentration of 3 at.% more than doubled the mass gain. In a separate study on Ni-20Cr alloys from 1000 to 1200 °C, Nagai et al. [19] also found the oxidation rate to increase with Ti concentration, which was attributed to an external NiO scale and accelerated growth of a Cr_2_O_3_ scale. They also observed that larger Ti concentrations resulted in significant levels of spallation and poor oxide scale adherence, which was suggested to be due to TiO_2_ dissolving in the oxide scale and subsequently increasing the vacancy concentration of Cr_2_O_3_. This effect was also observed by Cruchley et al. [20] in the commercial Ni-based superalloy RR1000 where sub-parabolic oxidation kinetics were attributed to a Ti-doped Cr_2_O_3_ scale. While the oxidation rates were significantly higher than Ti-free Cr_2_O_3_, the authors observed that the accelerated oxidation rates reduced after a prolonged period due to the eventual depletion of Ti in the alloy substrate. The findings of Barrett et al. [5] and Smialek et al. [2, 6] also reported the adverse effects of Ti on oxidation behaviour and showed the alloys with the best oxidation resistance to have relatively low concentrations of Ti. However, it has been shown in the literature that the addition of Ti can also have beneficial effects on oxidation resistance in certain circumstances. For example, Zhu et al. [21] reported that the addition of Ti significantly improved the oxidation performance of Co–Ni-based superalloys where Ti hindered the outward diffusion of Cr and promoted dense scales of TiO_2_ and Cr_2_O_3_.

The literature shows that Nb, Ta, and Ti can have significant yet mixed effects on the oxidation behaviour of Ni-based superalloys. It is therefore crucial to understand how these elements will affect oxidation phenomena when designing compositions for new Ni-based superalloys. To this end, the oxidation performance of the recently developed polycrystalline Ni-based superalloy C19 [22, 23] was assessed against systematic compositional variations of Nb, Ta, and Ti. The results were used to identify potential compositional adjustments to C19 for improved oxidation resistance.

## Experimental Procedures

### Materials

The Ni-based superalloy C19, which has been described previously [24], was used as a baseline for the creation of the alloys investigated in this study (Table 1). In this alloy, a relatively high concentration of Co was selected for solid solution strengthening, minimisation of stacking fault energy, and maintaining a low *γ*′ solvus. Nb and Ta were added for improved *γ*′ strengthening, while a relatively low Ti concentration was chosen to minimise excessive oxidation effects. A significant concentration of Mn was also selected for enhanced oxidation resistance. The thermodynamic software, *ThermoCalc 2021b,* was used to model the behaviour of several alloy parameters (e.g. solvi, APB energy, phase distributions) with systematic variations in the concentrations of Nb, Ta, and Ti. The findings enabled the identification of alloy concentrations that would maximise alloy performance while minimising the formation of topologically close-packed (TCP) phases and maintaining amenable levels of processability. Test samples with lower and higher concentrations of Nb, Ta, and Ti were selected for this study. These compositional modifications were accommodated by varying the concentration of Al to compensate such that the overall concentration of *γ*′ forming elements remained the same as that of C19. The intention of preparing two samples with a significantly large variation was to facilitate the identification of the effects of Nb, Ta, and Ti on the oxidation behaviour of the C19-based alloys.

**Table 1:** Nominal and actual compositions of the studied alloys with C19 provided for reference (at.%)

Element	C19	Nb1		Nb2		Ta1		Ta2		Ti1		Ti2	
	Nom	Nom	Act	Nom	Act	Nom	Act	Nom	Act	Nom	Act	Nom	Act
Al	9.41	10.43	10.34	9.67	9.48	11.00	10.89	9.82	9.72	11.30	11.31	9.89	9.83
Co	19.25	19.25	19.80	19.25	19.48	19.25	19.73	19.25	19.73	19.25	19.65	19.25	19.68
Cr	13.18	13.18	13.11	13.18	13.18	13.18	13.00	13.18	13.10	13.18	13.09	13.18	13.04
Mn	0.59	0.59	0.62	0.59	0.64	0.59	0.63	0.59	0.63	0.59	0.62	0.59	0.62
Mo	1.79	1.79	1.75	1.79	1.77	1.79	1.89	1.79	1.77	1.79	1.74	1.79	1.76
Nb	1.02	0.01	0.02	0.77	0.72	1.02	1.00	1.02	0.93	1.02	0.91	1.02	0.89
Ta	1.63	1.63	1.78	1.63	1.81	0.06	0.26	1.24	1.40	1.63	1.80	1.63	1.81
Ti	1.91	1.91	1.84	1.91	1.89	1.91	1.93	1.91	1.89	0.04	0.03	1.45	1.43
W	1.25	1.25	1.47	1.25	1.47	1.25	1.46	1.25	1.48	1.25	1.48	1.25	1.47
Zr	0.06	0.06	0.07	0.06	0.07	0.06	0.17	0.06	0.09	0.06	0.04	0.06	0.06
B	0.20	0.20	(0.20)	0.20	(0.20)	0.20	(0.20)	0.20	(0.20)	0.20	(0.20)	0.20	(0.20)
C	0.15	0.15	(0.15)	0.15	(0.15)	0.15	(0.15)	0.15	(0.15)	0.15	(0.15)	0.15	(0.15)
Ni	49.56	49.56	49.21	49.56	49.49	49.56	49.05	49.56	49.25	49.56	49.34	49.56	49.41

The alloys were prepared by melting elements of 99.9% purity or higher under vacuum with an Edmund Bühler Arc Melter. After melting, the ingots were sealed in glass tubes under an inert Ar atmosphere. The sealed ingots were subsequently subjected to super-solvus heat treatments for 6 h. The temperatures for the super-solvus heat treatments were selected to be between the *γ*′ solvus and solidus temperatures, which were determined using differential scanning calorimetry (DSC). The Ta1 and Ti2 alloys were super-solvus solutioned at 1180 °C and 1190 °C, respectively. The Nb2 and Ta2 alloys were super-solvus solutioned at 1185 °C, while the Nb1 and Ti1 alloys were super-solvus solutioned at 1200 °C. After solutioning, the sealed ingots were air-cooled and subjected to a commercial precipitate-ageing heat treatment at 843 °C and 800 °C for 2 h each.

The actual compositions of the investigated alloys were subsequently measured with large-area energy dispersive X-ray (EDX) analysis and averaged over three representative regions with approximate field-of-view dimensions of 52 × 40 μm^2^ and an acquisition time of 10 min per region (Table 1). EDX analysis was performed on the heat-treated alloys from which no evidence of significant chemical segregation was observed. This suggested that the selected super-solvus temperatures and duration of 6 h were suitable for achieving adequate homogeneity in the investigated alloys.

### Thermo-Gravimetric Analysis and Furnace Exposure Oxidation Experiments

Thermo-gravimetric analysis (TGA) measurements of the alloys were performed using a Setaram Setsys Evolution 18 apparatus to study the specific mass changes associated with oxidation. The TGA experiments were carried out at 800 °C for 100 h in air. TGA samples were prepared from each alloy with approximate 13 × 9 × 1 mm^3^ dimensions. Stereographs of the sample faces and thickness measurements were acquired and analysed with the image processing software ImageJ to obtain the sample surface areas so that specific mass changes could be accurately calculated from associated mass change data. All faces and sides of the TGA samples were polished to a 1 µm diamond finish. Before the TGA experiments, the samples were cleaned in acetone followed by ethanol in an ultrasonic bath and dried for a minimum of 24 h in a drying cabinet. The TGA experiments consisted of three sections: heating, isothermal, and cooling. In the heating section, the samples were heated from 20 to 200 °C at 10 °C/min and allowed to stabilise for 10 min. The samples were then heated from 200 to 775 °C at 20 °C/min. To avoid overshooting the target temperature, the samples were heated from 775 to 800 °C at 5 °C/min. In the isothermal section, the samples were held at 800 °C for 100 h. During the cooling section, the samples were cooled from 800 to 20 °C at 35 °C/min. To determine the oxidation behaviour of the studied alloys, the TGA data acquired throughout the full isothermal sections were fitted to a generalised form of the classical power law equation with the MATLAB Curve Fitting Tool:

1 $${\left(\Delta m\right)}^{n}={k}_{n}t$$

where $\Delta m$ is the specific mass change (mg/cm^2^), ${k}_{n}$ is the oxidation rate constant (mg^*n*^/cm^2*n*^h), and $t$ is the time (h). Parabolic oxidation behaviour is indicated by an $n$ ≈ 2 while “sub-parabolic” and “super-parabolic” behaviours are characterised by $n$ < 2 and $n$ > 2, respectively.

Furnace exposure oxidation experiments were carried out on the samples to investigate long-term oxidation effects. The samples used were elliptical cross sections of the arc-melted alloys with approximate 13 × 9 × 2 mm^3^ dimensions that were polished to a 1 µm diamond finish. The samples were individually exposed to air at 800 °C for 1000 h in a Carbolite CWF1100 laboratory furnace.

### Oxide Analyses

X-ray diffraction (XRD) data were acquired to detect and identify surface oxides on selected samples using a Bruker D8 ADVANCE fixed sample X-ray diffractometer fitted with a LynxEye EX position-sensitive detector. A standard programme (fixed illuminated length) was used to acquire diffraction data from the sample over a 2*θ* range of 10–80° with a step size of 0.02° and a dwell time of 1.5 s/step.

Samples for oxide examination were sectioned with a precision saw utilising a SiC blade, using a low-impact dry-cut method to minimise potential damage and preserve oxide layers on the sample surfaces. The sectioned samples were mounted in phenolic resin and ground down by 1 mm using SiC grinding papers to remove any potential cutting damage. Samples were polished with a 0.06 µm colloidal silica suspension before analysis.

Microstructural examination of the polished cross sections was carried out in a Zeiss GeminiSEM 300 scanning electron microscope (SEM) operated at 20 kV and a working distance of approximately 8.5 mm. The instrument was also equipped with an Oxford Instruments energy dispersive X-ray (EDX) detector to facilitate compositional analyses of microstructural features. Oxide cross sections were examined in backscattered electron (BSE) mode and EDX elemental concentration maps were concurrently acquired with BSE micrographs.

A general Rietveld analysis was performed on the XRD data using TOPAS-Academic V6 software [25] for oxide identification and phase fraction calculation. Appropriate CIF files were acquired from the Inorganic Crystal Structure Database (ICSD) through the Physical Sciences Data-Science Service (PSDS) [26]. Once a satisfactory preliminary fit was achieved, its weighted profile *R*-factor (*R*_wp_) was used as a metric for further refinement of the fit.

## Results

### Thermo-Gravimetric Analysis

**Fig. 1:**
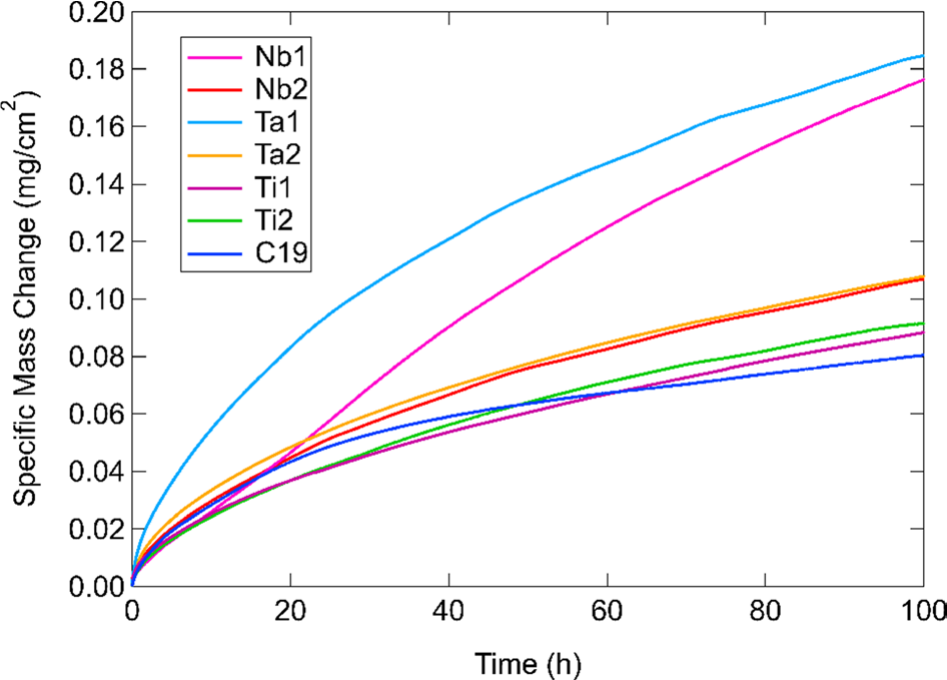
TGA specific mass change curves for the investigated alloys during oxidation at 800 °C for 100 h in air. The data for C19 measured in [24] are included for reference

The specific mass changes recorded using TGA for the investigated alloys during isothermal oxidation at 800 °C for 100 h are presented in Fig. 1. For reference, the equivalent data previously acquired for C19 [24] are also included. The Ti1 and Ti2 alloys exhibited the lowest overall specific mass changes (0.085 mg/cm^2^), while the Ta1 and Nb1 alloys showed the largest specific mass changes (0.180 mg/cm^2^). The Nb2 and Ta2 alloys exhibited relatively intermediate levels of specific mass change (0.100 mg/cm^2^). Except for Nb1, all alloys showed parabolic mass gain curves from the onset. In contrast, Nb1 displayed a pseudo-linear relationship for the majority of the 100 h. All alloys reached half of their final specific mass changes after approximately 20 h. The Nb2 alloy had a substantially decreased final specific mass change compared to Nb1. Similarly, the Ta2 alloy had nearly half of the final specific mass change of Ta1. The final specific mass changes of Ti1 and Ti2 showed relatively little difference.

The results in Fig. 1 were fitted to Eq. (1) for quantitative comparisons using the MATLAB Curve Fitting Tool. The fitted coefficients are outlined in Table 2 with 95% confidence bounds calculated by Student’s cumulative t-distribution function.

**Table 2:** Values of $n$ and ${k}_{n}$ from Eq. (1) for the investigated alloys after TGA exposure at 800 °C for 100 h with 95% confidence bounds in parentheses

Sample	800 °C/100 h	
	$n$	${k}_{n}$(g^*n*^/cm^2*n*^s)
Nb1	1.265 (1.263, 1.266)	3.225 × 10^−10^ (3.211, 3.236 × 10^−10^)
Nb2	1.848 (1.845, 1.852)	4.556 × 10^−11^ (4.511, 4.597 × 10^−11^)
Ta1	1.974 (1.968, 1.979)	1.034 × 10^−10^ (1.023, 1.044 × 10^−10^)
Ta2	1.993 (1.989, 1.997)	3.339 × 10^−11^ (3.308, 3.372 × 10^−11^)
Ti1	1.834 (1.832, 1.836)	3.247 × 10^−11^ (3.228, 3.267 × 10^−11^)
Ti2	1.757 (1.753, 1.761)	4.325 × 10^−11^ (4.278, 4.372 × 10^−11^)

### Cross-Sectional Analysis

**Fig. 2:**
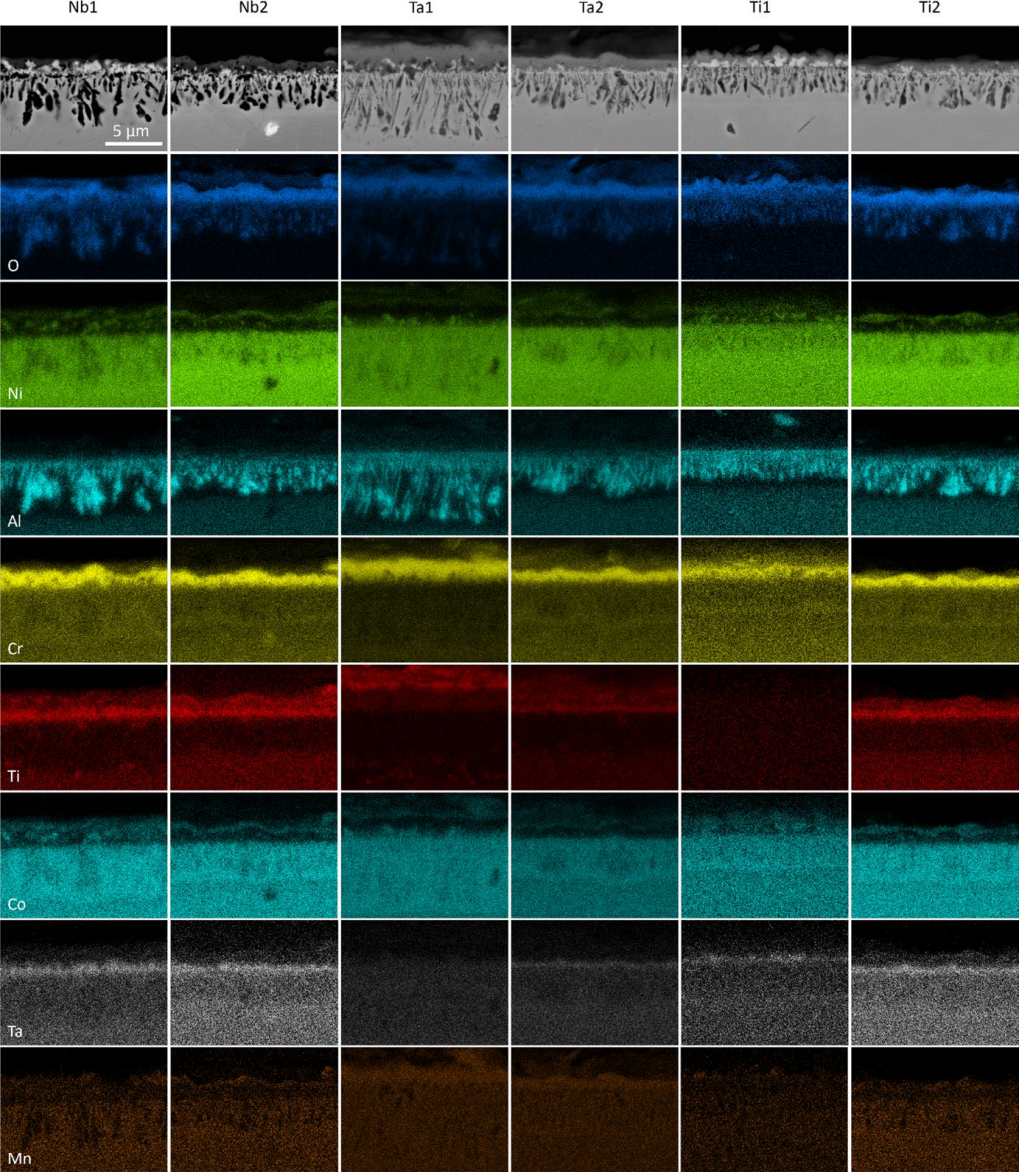
BSE SEM cross-sectional images (top row) and EDX elemental concentration maps for Nb1, Nb2, Ta1, Ta2, Ti1, and Ti2 alloys after oxidation in air at 800 °C for 1000 h

The BSE cross-sectional images and associated EDX elemental concentration maps for the alloys studied at 800 °C are presented in Fig. 2. All samples showed a thin external scale underpinned by discontinuous protuberances rich in Ni and Co, which were most prominent in Nb1 and Ti1. Significant concentrations of Cr were detected in the external scale with similar thicknesses between Nb1/Nb2 and Ti1/Ti2. However, the external Cr-rich scale in Ta2 appeared thinner compared to Ta1. Dark BSE-contrast discontinuous intrusions, associated with high Al content, were observed underneath the Cr-rich external scale in all samples. Nb1 and Ta1 showed the longest intrusions, while Nb2, Ta2, Ti1, and Ti2 had relatively similar intrusion lengths. Notably, the Al-rich intrusions in Ta1 showed long and thin “needle-like” morphologies that were thinner than the other samples and significantly longer than in Ta2. In all cases, an Al-depleted zone was observed beneath and surrounding the intrusions. An “internal” scale enriched in Ta and Ti was detected between the Cr-rich external scale and Al-rich intrusions in Nb1, Nb2, and Ti2. Ta1 showed significant Ti concentrations in the external scale, but no evidence of a Ta-rich internal layer was observed. Similarly, Ta2 and Ti1 showed relatively weak concentrations of Ta in the internal scale, with Ti1 being the only alloy where no prominent concentrations of Ti were observed in the external scale. Furthermore, all samples showed Mn-rich regions in the external oxide scale. In particular, Ta1 and Ta2 showed a relatively high concentration of Mn throughout the oxide scale while Ti1 showed relatively low concentrations of Mn in the oxide scale. The elemental maps of Nb, Mo, and W showed relatively uniform distributions (refer to Supplementary Information).

**Fig. 3:**
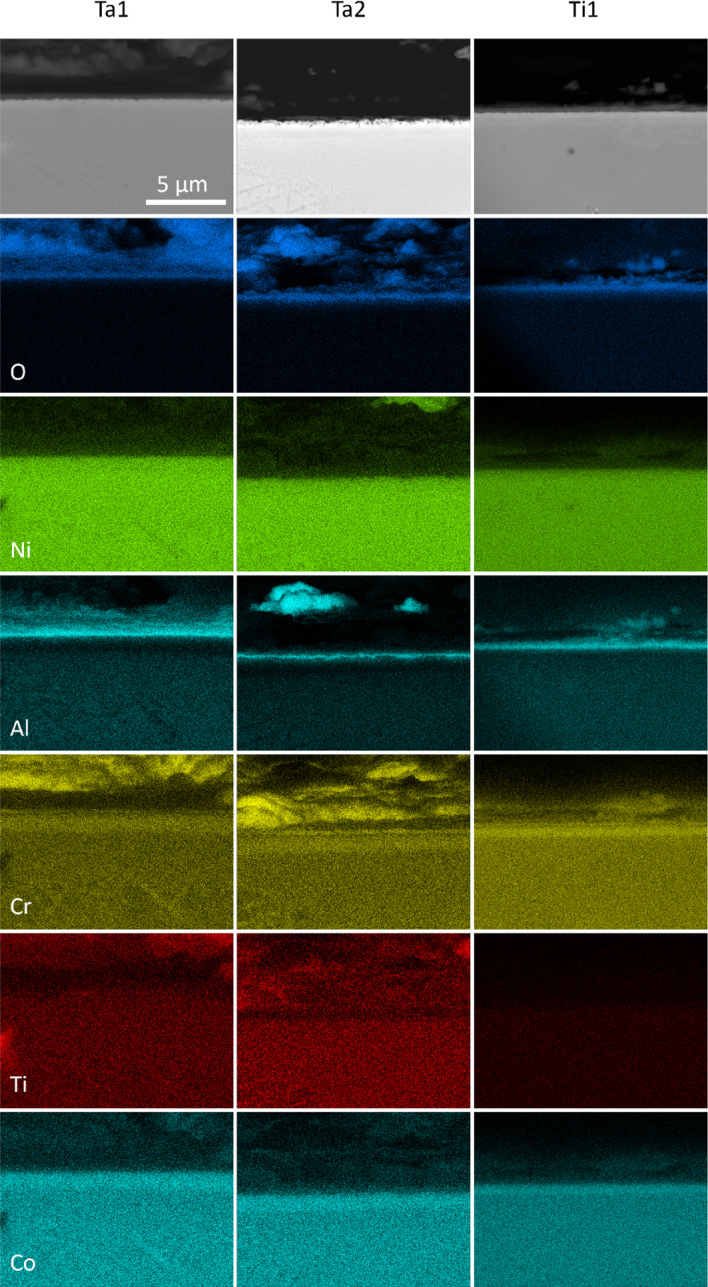
BSE SEM cross-sectional images (top row) and EDX elemental concentration maps of continuous Al-rich oxide regions in Ta1, Ta2, and Ti1 alloys after oxidation in air at 800 °C for 1000 h

In the investigated alloys, Ta1, Ta2, and Ti1 showed regions of uniform Al-rich scales on the surface after being exposed to 800 °C for 1000 h. The BSE cross-sectional images and EDX elemental concentration maps obtained from such regions are given in Fig. 3. All three alloys displayed thin and compact external scales enriched in Al and O. Beneath the Al-rich scale, Ta1 and Ta2 displayed an Al-depleted zone, while Ti1 did not show significant Al depletion. Non-Al elements showed no notable enrichment at the alloy-oxide interface and were uniformly distributed throughout the alloy substrate. In Ta1, a Ti-rich site was observed at the left of the Ti elemental map which coincided with increased B concentration. The Ta2 sample, imaged at a slightly angled perspective that made part of the top portion of the external scale visible, revealed protrusions rich in Ni, Al, Cr, and Ti. Ti1 demonstrated similar elemental distributions to Ta1 and Ta2 but exhibited weaker Ti concentrations in the alloy substrate.

### XRD Analysis

**Fig. 4:**
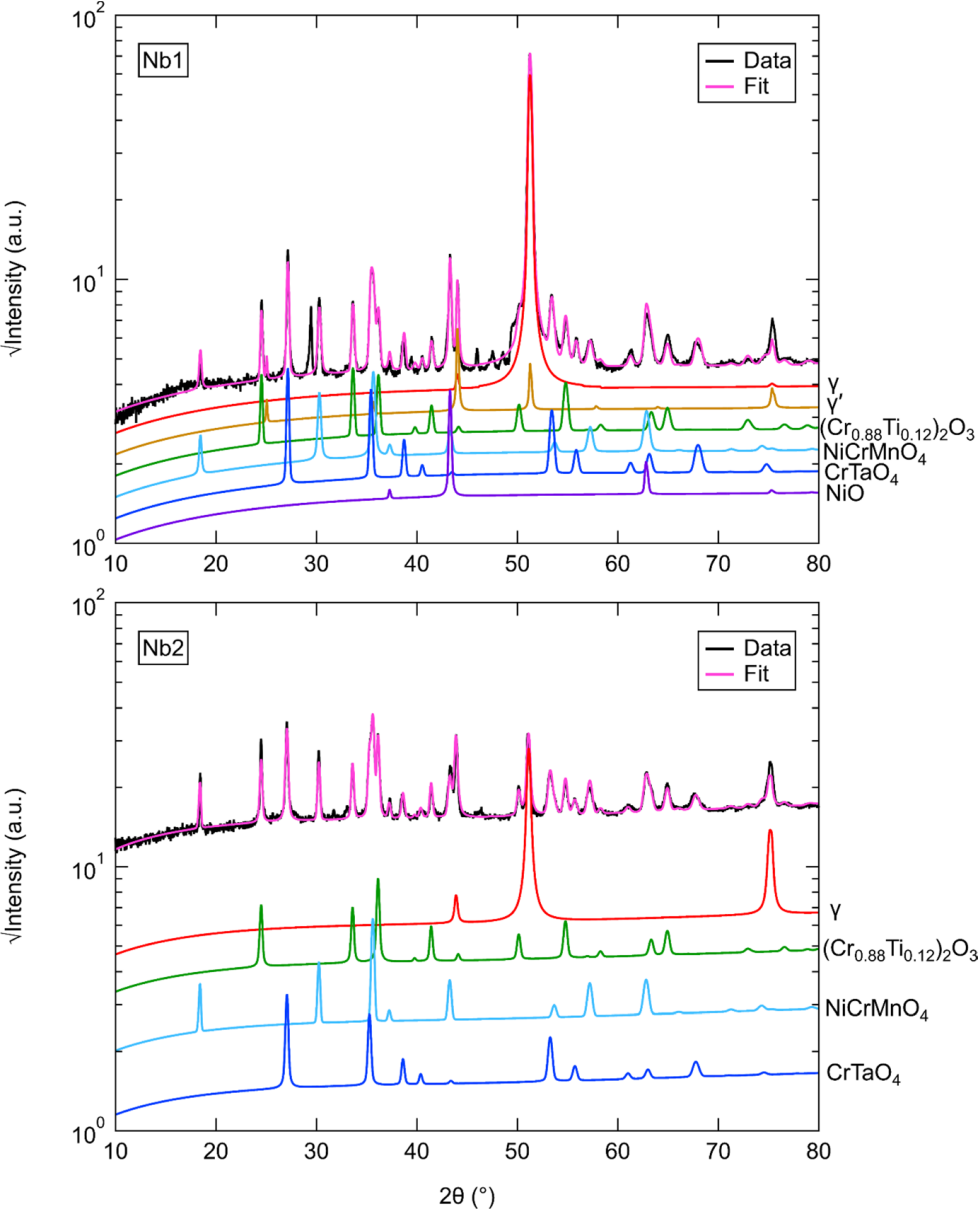
X-ray diffractograms for surfaces of the Nb1 and Nb2 alloys after oxidation at 800 °C for 1000 h in air. The simulated fits using Rietveld refinement with constituent phases are also shown

**Fig. 5:**
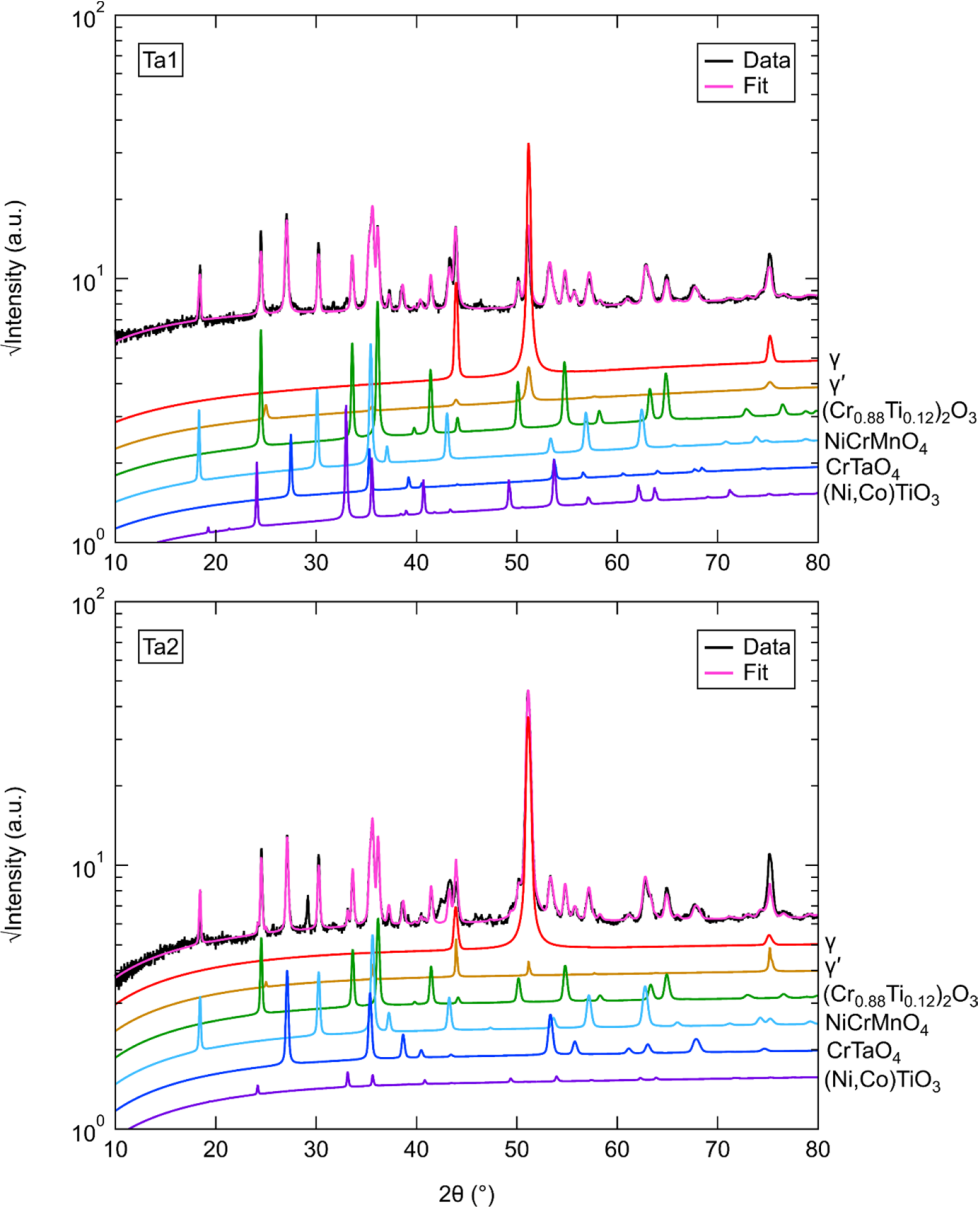
X-ray diffractograms for surfaces of the Ta1 and Ta2 alloys after oxidation at 800 °C for 1000 h in air. The simulated fits using Rietveld refinement with constituent phases are also shown

**Fig. 6:**
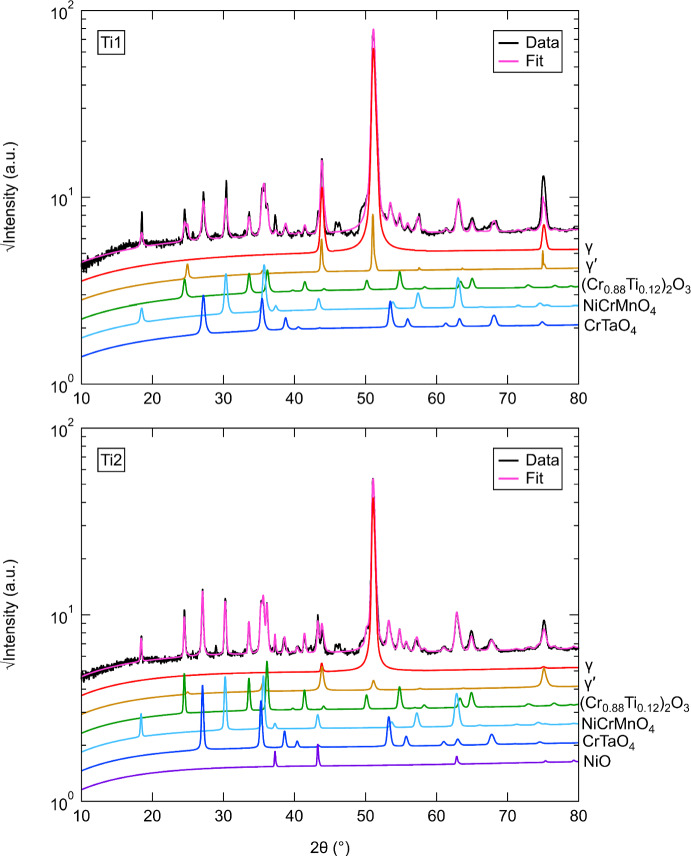
X-ray diffractograms for surfaces of the Ti1 and Ti2 alloys after oxidation at 800 °C for 1000 h in air. The simulated fits using Rietveld refinement with constituent phases are also shown

XRD diffractograms of the surface oxides from the alloys studied after oxidation at 800 °C for 1000 h are shown in Figs. 4, 5 and 6 (log scale). All alloys showed main diffraction peaks corresponding to *γ*, (Cr_0.88_Ti_0.12_)_2_O_3,_ NiCrMnO_4_, and CrTaO_4_. Nb1 showed peaks at approximately 45.9 and 48.5° that could not be identified as well as generally higher peak intensities for (Cr_0.88_Ti_0.12_)_2_O_3_ and CrTaO_4_ than Nb2. In contrast, Nb2 showed no evidence of *γ*′ and NiO. Both Ta1 and Ta2 displayed peaks for (Ni,Co)TiO_3_, with Ta2 exhibiting generally reduced peak intensities compared to Ta1 in the 20–45° angle range. Differences between the Nb and Ta datasets were observed: NiO was observed only in Nb1 while (Ni,Co)TiO_3_ was observed only in Ta1 and Ta2. However, both datasets contained peaks from *γ*, *γ*′, (Cr_0.88_Ti_0.12_)_2_O_3_, NiCrMnO_4_, and CrTaO_4_. Peak intensities of *γ* and *γ*′ were generally higher in Nb1 compared to Ta1, while the opposite was observed for Nb2 compared to Ta2. Additionally, slightly higher peak intensities of (Cr_0.88_Ti_0.12_)_2_O_3_ were observed in Nb1 and Ta1 compared to Nb2 and Ta2. Nb1 and Ta2 both showed a peak at approximately 29.4° that could not be identified. While Ti1 and Ti2 had similar overall peak intensities, NiO was identified in Ti2 but was absent in Ti1. A minor peak at approximately 28.9° was detected in Ti2 that did not match other examined phases; it was unclear if this peak was present in Ti1 or obscured by the background. Differences between the Ta and Ti datasets were observed. (Ni,Co)TiO_3_ was detected in Ta1 and Ta2 but absent in Ti1 and Ti2. The *γ* and *γ*′ peak intensities in Ta1 were generally lower than those in Ti1, while Ta2 and Ti2 showed similar intensities for these peaks. Ta1 had higher (Cr_0.88_Ti_0.12_)_2_O_3_ peak intensities compared to Ta2, Ti1, and Ti2. Differences between the Nb and Ti datasets were also noted, with evidence of NiO formation in Nb1 and Ti2 but not in Nb2 and Ti1. Nb2 showed the lowest *γ* peak intensities compared to Nb1, Ti1, and Ti2. Overall, the oxide intensities in the Nb and Ti datasets were similar. The unidentified peaks at approximately 28–29° in all samples and 45.9/48.5° in Nb1 were further investigated by repeating XRD analysis on unoxidized samples of the investigated alloys. The 28–29° peak was also detected in the unoxidized samples and could not be unambiguously indexed with any possible phases expected to be present, suggesting that it does not arise from the oxidation process. For Nb1, the minor peaks at 45.9° and 48.5° were not detected in the unoxidized state. The origin of these peaks is postulated to be from a minor phase present in the samples due to the relatively low peak intensities compared to other oxide peaks. However, the locations of these peaks are sufficiently separated from other oxide peaks such that the identification of the predominant oxides was not adversely impacted.

The phases and their respective weight fractions (wt.%) that were identified in the investigated alloys with XRD after oxidation at 800 °C for 1000 h are summarised in Table 3 for clarity. In addition, the associated uncertainties calculated with TOPAS-Academic V6 and the weighted profile *R*-factor (*R*_wp_) for each Rietveld refinement are shown. It is noted that the weight fractions of *γ*′ in Table 3 indicate the amounts of *γ*′ that were obtained with Rietveld refinement of the XRD data. However, as the interaction volumes for each sample include different proportions of the substrate and oxide scale in line with the oxide thickness, the weight fractions are not representative of the amount of *γ*′ in the bulk of the alloys. To minimise differences in the XRD interaction volume, each investigated alloy was placed in the XRD sample holders as identically as possible such that the incident X-ray beam contacted the samples at the same geometric locations.

**Table 3:** Weight fractions (wt.%) of identified phases in the investigated alloys at 800 °C and 1000 h

Phase (wt.%)	Nb1	±	Nb2	±	Ta1	±	Ta2	±	Ti1	±	Ti2	±
*γ*	11	8	20.4	1.1	24.3	0.6	22.2	0.1	60.5	1.8	9	4
*γ*′	9.4	1.5	–	–	2.1	0.9	3.2	0.5	5.4	1.3	9.7	1.3
(Cr_0.88_Ti_0.12_)_2_O_3_	24	3	34.6	1.8	34.4	0.6	32.6	0.2	9.7	1.5	29	2
NiCrMnO_4_	33	7	31.2	1.9	21.7	0.7	29.7	0.2	18.8	1.3	34	3
CrTaO_4_	14	1.9	13.8	1.0	2.1	0.2	10.2	0.1	5.6	0.5	15.4	1.1
NiO	9	4	–	–	–	–	–	–	–	–	3.5	1.1
(Ni,Co)TiO_3_	–	–	–	–	15.4	0.5	2.1	0.0	–	–	–	–
*R*_wp_	13.2		14.8		6.6		7.5		11.8		8.2	

The uncertainties and *R*_wp_ values for each Rietveld refinement are also shown and were calculated in TOPAS-Academic V6

## Discussion

### General

The presence of NiCrMnO_4_ was detected with XRD in relatively similar phase fractions in all the investigated alloys. However, NiCrMnO_4_ is not a commonly observed oxide in studies of similar alloys. In a work by Lee et al. [27], minor fractions of NiCrMnO_4_ were identified during the oxidation of Inconel 740 in air and Ar-based atmospheres, which was attributed to the oxidation of Mn and Cr into the NiCrMnO_4_ spinel. In their study, NiCrMnO_4_ was not observed to form at 800 °C but did so between 900 and 1000 °C. Similarly, Hussain et al. [28] observed the presence of NiCrMnO_4_ as well as other oxides in Hastelloy C-4 after exposure to steam from 600 to 1200 °C for 1–400 h. It is possible that the concentration of Mn in the alloys investigated in the present work led to a Mn-rich external scale (Fig. 2) which coexists with the Cr_2_O_3_ scale. We have previously identified a similar Mn enrichment effect at the external scale in the Ni-based superalloy C19 [24]. It is noted that increasing the Ta and Ti concentrations, as evidenced for Ta1 and Ta2 and Ti1 and Ti2, appears to also increase the phase fraction of NiCrMnO_4_, suggesting that Ta and Ti additions may have similar effects on the formation of NiCrMnO_4_. In contrast, Nb concentration has relatively little effect on the amount of NiCrMnO_4_ formed. Due to the relative lack of data for the formation of NiCrMnO_4_ during the oxidation of Ni-based superalloys, further investigation of alloys derived from the Ni–Cr–Mn ternary system could improve understanding of its origins and oxidation behaviour.

The existence of an internal scale enriched in Ta directly beneath the Cr_2_O_3_ scale was observed in all the investigated alloys except Ta1. This Ta-rich internal scale is putatively identified as CrTaO_4_ from the XRD and EDX data. This phase was also reported in [7], and its occurrence was shown to be promoted by the addition of Nb, which increased the activities of Cr and Ta. This explanation is consistent with the results observed in both Nb1 and Nb2, which exhibited the highest phase fractions of CrTaO_4_. In contrast, the relatively low Ta concentration in Ta1 led to nearly no CrTaO_4_, as shown in Fig. 2. This is further supported by the Rietveld analysis, which showed that Ta1 had the lowest CrTaO_4_ phase fraction. Contrary to other studies [29, 30], no evidence of Ta_2_O_5_ was observed in any of the investigated alloys. However, it is possible that Ta_2_O_5_ may have formed as an initial oxide and subsequently reacted with Cr_2_O_3_ to form CrTaO_4_ through the following solid-state reaction:

$${{\text{Cr}}}_{2}{{\text{O}}}_{3} \left(s\right)+{{\text{Ta}}}_{2}{{\text{O}}}_{5} \left(s\right)\to {2{\text{CrTaO}}}_{4} \left(s\right) {\Delta }_{f}{G}^{^\circ }=-457\frac{{\text{kJ}}}{\mathrm{mol }{{\text{O}}}_{2}}$$

where $s$ denotes a solid. The Gibbs free energy of formation ${\Delta }_{f}{G}^{^\circ }$ for CrTaO_4_ was calculated by interpolating ${\Delta }_{f}{G}^{^\circ }$ values extracted from tables in [31] for Cr_2_O_3_ and [32] for Ta_2_O_5_, respectively. Following the approach of Müller et al. [33], which is based on Massard et al. [34], the ${\Delta }_{f}{G}^{^\circ }$ values of Cr_2_O_3_ and Ta_2_O_5_ were used to calculate a weighted average ${\Delta }_{f}{G}^{^\circ }$ value of CrTaO_4_.

Of particular interest is the detection and formation of (Cr_0.88_Ti_0.12_)_2_O_3_ as it is not frequently reported in similar studies [20, 35] from 600 to 900 °C. However, some authors [36, 37] have reported its formation at 650–900 °C and attributed it to a Ti-doped Cr_2_O_3_ scale. In the investigated alloys, Ti1 had the lowest phase fraction of (Cr_0.88_Ti_0.12_)_2_O_3_, which is consistent with its low Ti concentration. To rationalise the formation mechanism of (Cr_0.88_Ti_0.12_)_2_O_3_, Blacklocks et al. [38] and Atkinson et al. [39] have proposed theories where TiO_2_ can dissolve into Cr_2_O_3_ such that Ti ions are substituted onto the Cr sites, creating excess vacancies for increased diffusion of Cr ions through the Cr_2_O_3_ scale and therefore enhanced oxidation rates. However, the relatively low Ti concentration in Ti1 (< 2 at.%) may have resulted in an insufficient volume of TiO_2_ that was not conducive for the defect reaction to proceed, which could explain its relatively low fraction of (Cr_0.88_Ti_0.12_)_2_O_3_. The findings of Barrett et al. [5] also suggested that Ni-based alloys with Ti concentrations under 2 at.% had the best oxidation resistance. However, the results reported in [14] showed a dramatic increase in oxidation rates at 3 at.%, suggesting that the Ti concentration of C19 should not be increased further.

### The Effect of Nb

Analysis of the TGA results in Fig. 1 suggests that the specific mass change decreases with increasing Nb concentration. This outcome is unexpected since several studies [6–9] have shown that the addition of Nb is typically detrimental to the oxidation resistance of a Ni-based superalloy. Furthermore, the respective fitted mass gain exponents $n$ for Nb1 and Nb2 are 1.265 and 1.848, which further highlights the dramatic difference between the two alloys. In the TGA data, the early oxidation (< 20 h) mass gain data of Nb1 suggest that it undergoes a prolonged transient oxidation period in which non-protective oxides are being formed. After 20 h, the specific mass change curve for Nb1 adopts a slightly more parabolic profile. In contrast, the Nb2 alloy experiences parabolic kinetics from the onset of oxidation, and this is evidenced by its $n$ value. Moreover, the ${k}_{n}$ value of Nb1 (1.161 × 10^−3^ mg^*n*^/cm^2*n*^h) is an order of magnitude larger than Nb2 (1.640 × 10^−4^ mg^*n*^/cm^2*n*^h). Despite the large difference in specific mass changes, the BSE and EDX results in Fig. 2 show relatively similar results. Both alloys formed a prominent (Cr_0.88_Ti_0.12_)_2_O_3_ external scale beneath which discontinuous Al_2_O_3_ intrusions were formed. However, the O map for Nb2 appears to be denser and more compact than the O map in Nb1. It is therefore possible that the external oxide scale in Nb1 could be less protective and more amenable to oxygen ingress than Nb2. A possible reason for this could be due to the longer transient oxidation phase observed in the TGA results for Nb1. This explanation is supported by the detection of NiO, commonly associated with non-protective oxidation [4], in the XRD data and the Rietveld analysis. However, there was no significant evidence of Co-based oxides in the XRD data despite the presence of Co-rich areas throughout the oxide scale. This is potentially due to the sensitivity limitations of conventional XRD techniques. In the EDX results for Nb2, the discontinuous oxide regions with elevated Ni and Co signals are less prominent than in Nb1 but seem to have formed a more continuous thin oxide scale on the top surface of the external scale.

Several studies have reported the addition of Nb to be beneficial for oxidation resistance in Ni-based superalloys [10, 11, 40]. In a Ni–Fe–Cr alloy based on Alloy 718, Kuo et al. [40] found that systematic additions of Nb resulted in reduced oxidation kinetics at 800 °C. They attributed this phenomenon to the formation of a continuous CrNbO_4_ layer that acted as a diffusion barrier to oxygen ingress and Fe/Ni egress, which subsequently facilitated the formation of a Cr_2_O_3_ scale. These results were further corroborated by Weng et al. [10] where an approximate 25% improvement in the oxidation rate constant was reported after the addition of Nb. However, the authors also stressed that excessive Nb addition could lead to exacerbated oxidation performance by forming discontinuous inner oxide layers that enhance oxygen ingress. In a similar study [11], the same authors also noted improved oxidation resistance of Ni-based superalloys at 800 °C with small additions of Nb combined with Y. Therefore, it is possible that the increased Nb concentration of Nb2 may have reduced the outward diffusion of Ni to the surface and hindered the ingress of oxygen such that a dense and protective Cr_2_O_3_ scale was formed. However, unlike the previous studies, there was no EDX evidence of a Nb-rich inner scale in either Nb1 or Nb2.

### The Effect of Ta

The TGA specific mass changes in Fig. 1 for Ta1 and Ta2 suggest that oxidation resistance is significantly improved with increasing Ta concentration. This finding is consistent with other reports in the literature [1, 5, 14] where Ta was shown to have a favourable effect on reducing oxidation rates. The TGA curves for both Ta1 and Ta2 exhibited strong parabolic profiles which were further confirmed by the fitted values for $n$ (1.974 and 1.993, respectively). The ${k}_{n}$ values for Ta1 and Ta2 were in the same order of magnitude (10^−4^), but the former showed a nearly tripled oxidation rate. It is unclear why Ta2 resulted in a lower overall specific mass change despite having a lower Al concentration relative to Ta1. The EDX results for Ta1 in Fig. 2 showed no evidence of any Ta-containing oxides, which contrasts with Ta2 where a scale of Ta-rich oxide formed underneath the Cr_2_O_3_ scale. The Ta-containing oxide in Ta2 may have acted as a diffusion barrier to oxygen ingress (which was also observed in the Nb1 and Nb2 alloys), thereby facilitating the formation of Cr_2_O_3_. This finding is similar to the work of Yang et al. [14] and Kawagishi et al. [41] where minor and moderate additions of Ta improved oxidation resistance by forming NiTa_2_O_6_. However, the role of Ta in the oxidation of Ni-based alloys is a subject of debate. For instance, Park et al. [17] found Ta to be detrimental to oxidation resistance which was attributed to Ta reducing the outward diffusion of Al and therefore preventing the formation of a continuous Al_2_O_3_ scale. However, the Ta1 and Ta2 alloys primarily relied on the formation of Cr_2_O_3_ as a protective scale instead of Al_2_O_3_, meaning that any potential hindering effects of Al associated with the increased Ta concentration in Ta2 (1.24 at.%) are unlikely to improve the oxidation response. The lack of a Ta-rich oxide layer could also explain why the discontinuous Al_2_O_3_ intrusions in Ta1 were longer and deeper than those observed in Ta2, suggesting that oxygen could have travelled further into the Ta1 alloy substrate. While there was no direct evidence of NiTa_2_O_6_ in the XRD patterns, peaks associated with CrTaO_4_ were detected, as discussed earlier. Unlike the other alloys studied, the (Ni,Co)TiO_3_ compound was only detected in Ta1 and Ta2. The generalised form (Ni,Co)TiO_3_ is used here as it was not possible to differentiate NiTiO_3_ from CoTiO_3_ due to their similar crystal structure and lattice parameters. This phase is seldom observed; however, it may result from a solid-state reaction between NiO and TiO_2_ [42]. Combined with CoO [43], the reaction can be described as follows:

$$({\text{Ni}},{\text{Co}})\mathrm{O }\left(s\right)+{{\text{TiO}}}_{2} \left(s\right)\to {\left({\text{Ni}},{\text{Co}}\right){\text{TiO}}}_{3} (s) {\Delta }_{f}{G}^{^\circ }=-9.63\frac{{\text{kJ}}}{\mathrm{mol NiTi}{{\text{O}}}_{3}}$$

where $s$ denotes a solid. The Gibbs free energy of formation ${\Delta }_{f}{G}^{^\circ }$ for NiTiO_3_ was calculated with the expression reported by Jacob et al. [44].

It is noted that both Ta1 and Ta2 had isolated regions where only continuous Al_2_O_3_ was observed (Fig. 3) despite being primarily Cr_2_O_3_-formers (Type II). It is unclear as to why this occurred in both alloys given that the primary difference was in the Ta and Al concentrations. However, it has been previously reported that the composition of the C19 alloy, from which Ta1 and Ta2 are derived, is in a transition region between Type II and Type III oxidation behaviour [24]. Since the compositions of Ta1 and Ta2 replaced Ta with Al, the larger Al concentrations may have resulted in a further drive towards Type III oxidation.

### The Effect of Ti

The systematic variation of Ti concentration in Ti1 and Ti2 had virtually negligible effects on the TGA specific mass changes in Fig. 1. Both alloys followed relatively parabolic oxidation kinetics and resulted in an approximate specific mass change of 0.085 mg/cm^2^ after 100 h. In addition, Ti1 and Ti2 shared similar values of $n$ and ${k}_{n}$. These results closely resemble the TGA curve for C19, suggesting that the variation of Ti did not have a notable effect on the oxidation rate of C19. This result was unexpected since conventional wisdom suggests that Ti is detrimental to oxidation performance [6, 19]. The results suggest that even an approximate 97% reduction in the Ti concentration in Ti1 (to 0.04 at.%) compared to Ti2 (1.45 at.%) had no observable effect on the oxidation behaviour of C19. EDX examination of Ti1 showed no evidence of TiO_2_, which is consistent with its relatively low Ti concentration (0.04 at.%). This contrasts with Ti2 where a significant scale of TiO_2_, as part of the Ti-doped (Cr_0.88_Ti_0.12_)_2_O_3_, was observed. Otherwise, both alloys formed similar oxide strata characterised by a prominent NiCrMnO_4_ external scale, Ni/Co surface oxides, discontinuous Al_2_O_3_ intrusions, and a Ta-enriched internal scale.

Isolated regions of continuous Al_2_O_3_ formation (Type III) in Ti1 are observed in Fig. 3 where Al was the only alloying element to form an oxide scale, which was also confirmed in the XRD data. The resulting Al_2_O_3_ scale was extremely thin (< 1 µm), and no evidence of discontinuous Al_2_O_3_ intrusions was detected. However, Ti2 showed no such behaviour and retained strong Type II oxidation behaviour. The lower Ti concentration in Ti1 and increased Al concentration of (11.30 at.%) is likely to have promoted external Al_2_O_3_ scale formation. Some evidence of oxide detachment as a potential result of post-oxidation handling is also visible in the EDX data, which shows augmented levels of Cr and Al as well as relatively minor levels of Mn, Co, and Ni. While this could be the remains of an external scale of Cr_2_O_3_ that formed during the oxidation process, it does not explain the absence of discontinuous Al_2_O_3_ intrusions which would otherwise also be expected to be present. Therefore, other factors may have been responsible for the regions of continuous Al_2_O_3_ observed in Ti1, and further investigation is needed to identify the cause (e.g. grain boundary diffusion effects).

In general, the results of Ti1 and Ti2 suggest that a significant proportion of the Ti in C19 could potentially be replaced by other alloying additions with fewer detrimental oxidation effects (e.g. Nb, Ta) for improved environmental resistance. However, it is anticipated that the total elimination of Ti could adversely affect the APB energy [45] and hot corrosion resistance [46]. Therefore, any adjustments to the Ti concentration in C19 must balance the competing needs of these material properties.

### The Combined Effect of Nb and Ta

**Fig. 7:**
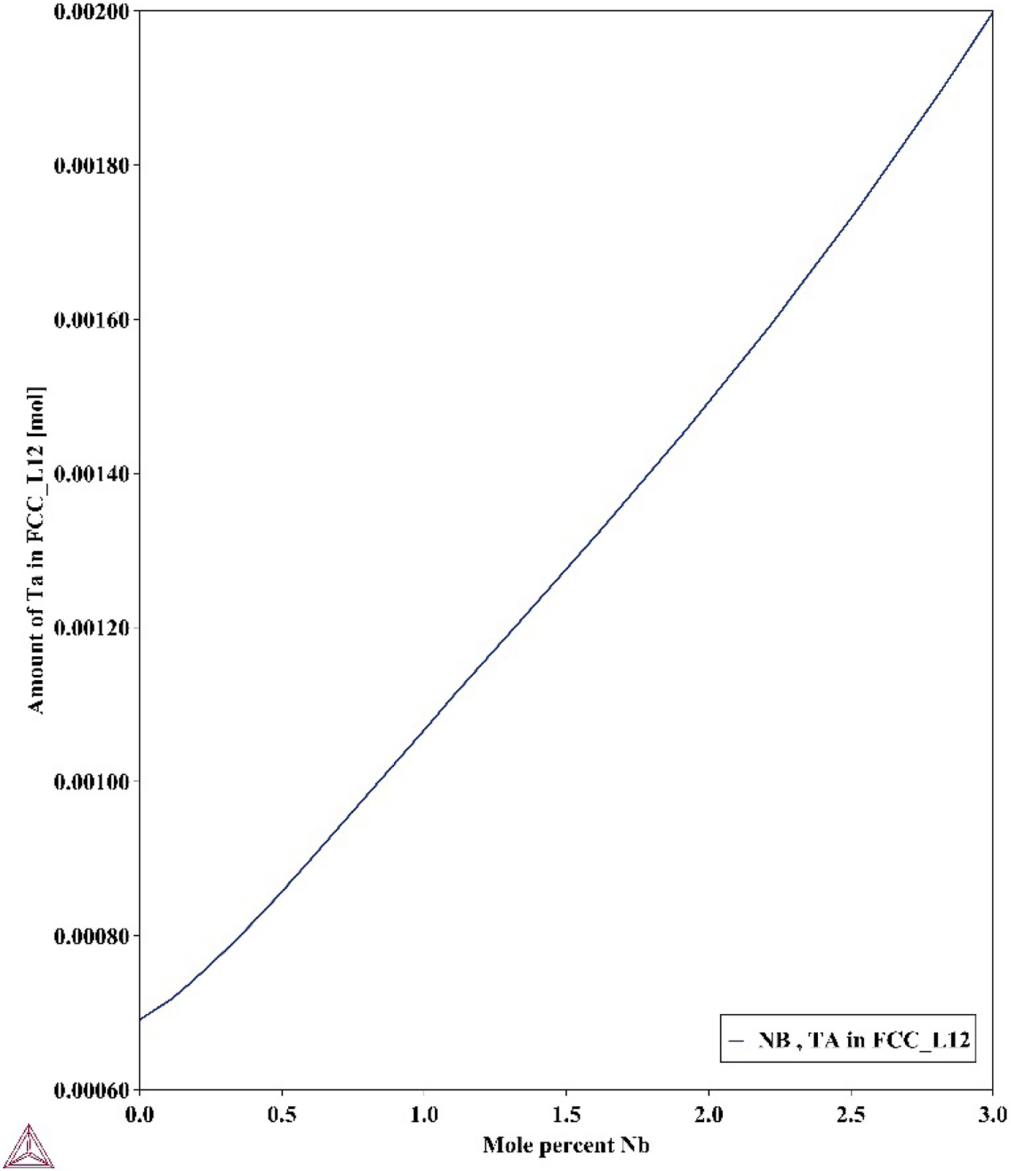
Ta concentration in the *γ* phase with increasing Nb concentrations at 800 °C. Calculated in ThermoCalc 2022b

The occurrence of CrTaO_4_ in alloys with different concentrations of Nb and Ta is of particular interest. Specifically, the absence of Nb-rich oxides in the oxide scales of the investigated alloys contrasts with the work reported in [40], which observed beneficial Nb-rich oxides that promoted Cr_2_O_3_ formation. Instead, the formation of a Ta-rich CrTaO_4_ internal scale suggests that the effect of Ta is more significant than that of Nb in the investigated alloys. It has been reported that increases in Nb can increase the activity of Ta in small increments [7], which may explain why the phase fractions of CrTaO_4_ in the Nb-lean Nb1 and Nb-rich Nb2 samples in Table 3 show negligible differences. However, the TGA results in Fig. 1 show that low concentrations of Nb and Ta (i.e. Nb1 and Ta1) resulted in poor oxidation resistance, while the samples with higher additions of both Nb and Ta resulted in notably better oxidation resistance. To rationalise this beneficial combined effect of Nb and Ta, modelling in ThermoCalc 2022b with the composition of C19 was carried out (Fig. 7). Systematic increases in Nb concentration led to significantly increased Ta concentration in the *γ* phase, which may have diffused to the alloy surface to form CrTaO_4_ due to its higher diffusivity in *γ* compared to *γ*′. Therefore, this result shows a potential mechanism through which the collaborative effect of both Nb and Ta may have led to the improved oxidation resistance in the investigated alloys.

The identification of (Cr_0.88_Ti_0.12_)_2_O_3_, as discussed earlier in this section, suggests the possibility of accelerated Cr_2_O_3_ formation by Ti-doping. However, the ${k}_{n}$ values in Table 2 show relatively similar values in the investigated samples except for Ta1. The similar ${k}_{n}$ values suggest that the concomitant effects of Nb and Ta additions in forming a CrTaO_4_ scale may dominate over any Ti-doping effects. This is further supported by Ta1 being the only investigated alloy that failed to form a continuous CrTaO_4_ layer, which may have led to its relatively large ${k}_{n}$ value. However, the effects of Ti may not be completely shielded from the formation of CrTaO_4_ since the phase fraction of CrTaO_4_ in Ti1 is significantly lower than in Ti2 despite both alloys having identical Nb and Ta concentrations. It is possible that the reduced Ti concentration in Ti1 may have reduced Ti-doping of Cr_2_O_3_, which slowed the oxidation rate of Cr. As a result, this may have reduced the amount of Cr_2_O_3_ available to react with Ta_2_O_5_ to form CrTaO_4_ in Ti1 compared to Ti2 for a given time of oxidation exposure.

It is noted that the compositional complexity of commercially relevant Ni-based superalloys, such as those investigated in this study, can present challenges in interpreting oxidation behaviour. Systematic studies with binary alloys may therefore be helpful for isolating the individual oxidation effects of Nb and Ta.

### The Formation of Continuous Al_2_O_3_

In Fig. 3, Ta1, Ta2, and Ti1 alloys formed regions of continuous Al_2_O_3_. To rationalise this behaviour, calculations were performed in ThermoCalc 2022b software with the composition of C19 to systematically model the activities and diffusivities of Al and Cr with increasing concentrations of Nb, Ta, and Ti. The tracer diffusion coefficient was used as a general metric for the diffusional capability of Al and Cr. The calculations showed that the activities and diffusivities of both Al and Cr decreased with increasing Nb, Ta, and Ti concentration, suggesting a hindering effect on the formation of continuous Al_2_O_3_. Similar findings were also obtained with ThermoCalc calculations in ternary Ni–Al–(Nb,Ta,Ti) systems. However, these predictions contrasted with the experimental results where an increased propensity to the formation of continuous Al_2_O_3_ was observed. These effects may be further compounded by the correlated effect between the activities of Al and Cr due to a known synergistic relationship between these two elements [4], making it challenging to isolate individual elemental contributions.

**Fig. 8:**
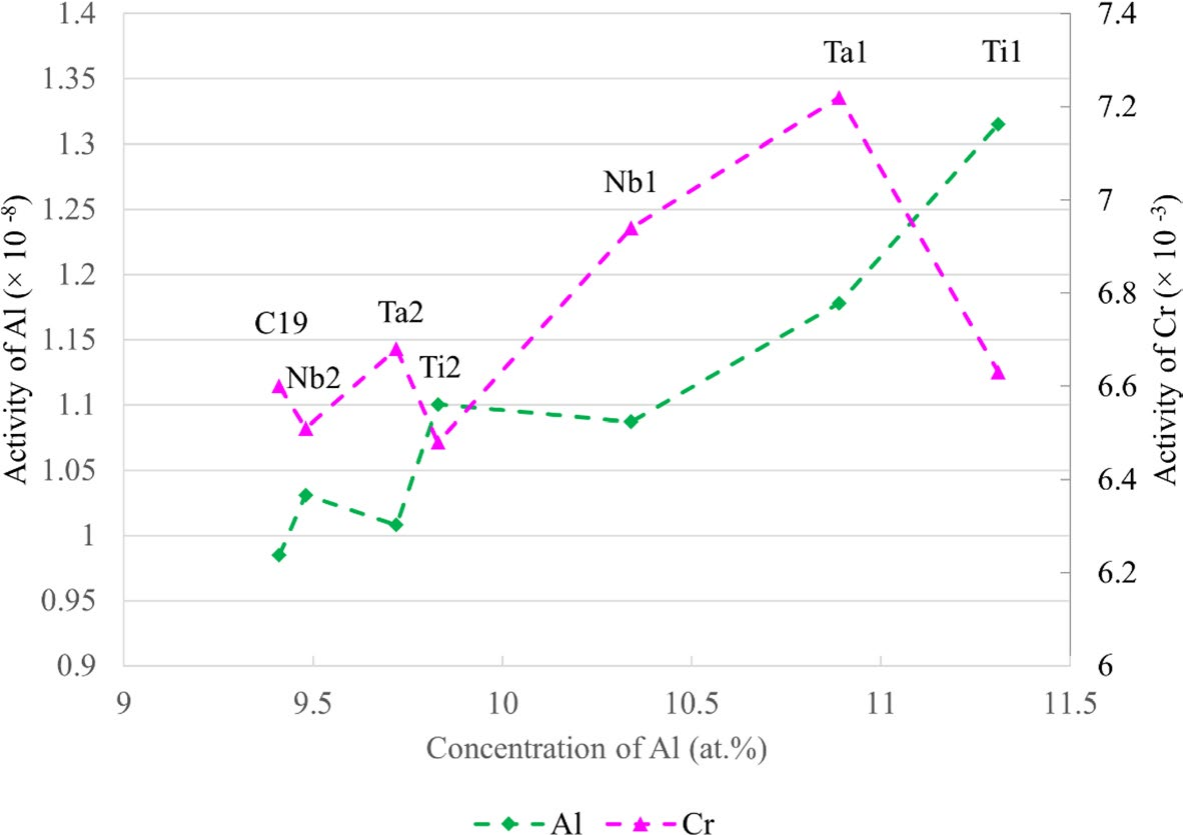
Activities of Al and Cr in C19, Nb1, Nb2, Ta1, Ta2, Ti1, and Ti2 alloys at 800 °C plotted against increasing Al concentration. Calculated in ThermoCalc 2022b

**Fig. 9:**
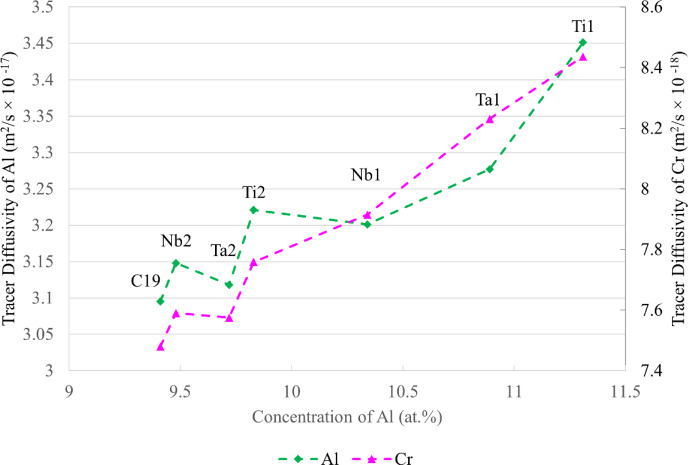
Tracer diffusion coefficients of Al and Cr in C19, Nb1, Nb2, Ta1, Ta2, Ti1, and Ti2 alloys at 800 °C plotted against increasing Al concentration. Calculated in ThermoCalc 2022b

The activities and diffusivities of Al and Cr in the studied alloys at 800 °C were computed with ThermoCalc 2022b and plotted in Figs. 8 and 9, respectively. It is apparent that two of the continuous Al_2_O_3_-forming alloys (Ta1 and Ti1) have the highest Al and Cr diffusivities. These alloys also have the highest activities of Al. In contrast, the remaining continuous Al_2_O_3_-forming alloy Ta2 exhibits lower activities and diffusivities of Al than Nb1 and Ti2 which did not form continuous Al_2_O_3_. It is unclear why Ta2 formed regions of continuous Al_2_O_3_. Further work is needed to elucidate the mechanisms of continuous Al_2_O_3_ formation in Ta1, Ta2, and Ti1. In this regard, similar experiments in model alloys, as discussed in the previous section, could help further rationalise the results in this study. However, it is still expected that the results observed herein may be useful for designing polycrystalline Ni-based superalloys that form continuous Al_2_O_3_ scales for oxidation resistance.

## Conclusions

In this study, the effect of systematic variations in Nb, Ta, and Ti concentration on the oxidation behaviour of the Ni-based superalloy C19 in air at 800 °C for up to 1000 h was investigated. The main findings are summarised as follows:

- All the studied alloys exhibited Type II oxidation behaviour by relying on a prominent external scale of Ti-doped (Cr_0.88_Ti_0.12_)_2_O_3_ for oxidation resistance. The presence of NiCrMnO_4_, CrTaO_4_, and NiO was also detected with SEM–EDX and XRD.
- The addition of Nb improved oxidation performance at 800 °C which may be attributed to the formation of a CrTaO_4_ layer that may act as a diffusion barrier to oxygen ingress and facilitate Cr_2_O_3_ scale growth. Contrary to conventional reports in the literature, the results suggest some Nb is beneficial for oxidation resistance in C19.
- The addition of Ta significantly improved oxidation performance at 800 °C and followed strong parabolic kinetics. Several regions of continuous Al_2_O_3_ formation in both alloys studied were attributed to higher Al concentrations because of substitution for Ta. The (Ni,Co)TiO_3_ oxide was also detected.
- The addition of Ti did not significantly affect oxidation at 800 °C, which contradicts other reports in the literature. The low Ti concentrations of the studied alloys may have avoided the detrimental effects on the oxidation rates reported in other studies. This suggests that partial or complete substitution of Ti in C19 with alloying elements (e.g. Nb, Ta) to improve other properties could be possible. Several regions of continuous Al_2_O_3_ formation were also observed and associated with higher Al concentrations.

### Supplementary Information

Below is the link to the electronic supplementary material.

Supplementary file1 (DOCX 15681 KB)

## Data Availability

The underlying research data required to reproduce these findings are available from the University of Cambridge repository [47]: https://doi.org/10.17863/CAM.97038.

## References

[1] Darolia R (2019). International Materials Reviews.

[2] J. L. Smialek, C. Barrett, and J. C. Schaeffer, *Design for Oxidation Resistance*. *The Materials Information Society* (1997), p. 20.

[3] Barrett CA, Lowell CE (1977). Oxidation of Metals.

[4] Giggins C, Pettit F (1971). Journal of the Electrochemical Society.

[5] Barrett CA, Miner RV, Hull DR (1983). Oxidation of Metals.

[6] Smialek JL, Bonacuse PJ (2016). Materials at High Temperatures.

[7] Ye X, Yang B, Nie Y, Yu S, Li Y (2021). Corrosion Science.

[8] Ye X, Yang B, Lai R, Liu J, Yu S, Li Y (2022). Corrosion Science.

[9] Alkmin LB, Chaia N, Utada S (2021). Metallurgical and Materials Transactions A.

[10] Weng F, Yu H, Chen C, Wan K (2015). Materials and Manufacturing Processes.

[11] Weng F, Yu H, Chen C, Wan K (2015). Surface and Interface Analysis.

[12] Bouillet C, Ciosmak D, Lallemant M, Laruelle C, Heizmann JJ (1997). Solid State Ionics.

[13] Ghosh G, Olson GB (2007). Acta Materialia.

[14] Yang S (1981). Oxidation of Metals.

[15] Lu X, Tian S, Yu X, Wang C (2011). Rare Metals.

[16] Guo H, Wang D, Peng H, Gong S, Xu H (2014). Corrosion Science.

[17] Park SJ, Seo SM, Yoo YS, Jeong HW, Jang HJ (2015). Corrosion Science.

[18] Nowak WJ, Wierzba B, Sieniawski J (2018). High Temperature Materials and Processes.

[19] Nagai H, Okabayashi M (1981). Transactions of the Japan Institute of Metals.

[20] Cruchley S, Evans HE, Taylor MP, Hardy MC, Stekovic S (2013). Corrosion Science.

[21] Zhu Y-X, Li C, Liu Y-C, Ma Z-Q, Yu H-Y (2020). Journal of Iron and Steel Research International.

[22] M. C. Hardy, K. Christofidou, P. M. Mignanelli, H. J. Stone, N. G. Jones, and C. Argyrakis, *Nickel-Base Superalloy US Patent No. 0360077* (2019), pp. 1–6.

[23] M. C. Hardy, K. Christofidou, P. M. Mignanelli, H. J. Stone, N. G. Jones, and C. Argyrakis, *Nickel-Base Superalloy US Patent No. 0360078* (2019), pp. 1–6.

[24] Wo JWX, Pang HT, Wilson AS, Hardy MC, Stone HJ (2022). Metallurgical and Materials Transactions A.

[25] Coelho AA (2018). Journal of Applied Crystallography.

[26] FIZ Karlsruhe GmbH, *Inorganic Crystal Structure Database (ICSD)* (1985).

[27] Lee DB, Kim MJ (2021). Journal of the Korean Institute of Surface Engineering..

[28] Hussain N, Qureshi AH, Shahid KA, Chughtai NA, Khalid FA (2004). Oxidation of Metals.

[29] Rehman K, Sheng N, Sang Z (2021). Vacuum.

[30] Hu YB, Cheng CQ, Cao TS, Zhang L, Zhao J (2022). Corrosion Science.

[31] Barin I (1995). Thermochemical Data of Pure Substances.

[32] Jacob KT, Shekhar C, Waseda Y (2009). The Journal of Chemical Thermodynamics.

[33] Müller F, Gorr B, Christ HJ (2019). Corrosion Science.

[34] Massard P, Bernier JC, Michel A (1972). Journal of Solid State Chemistry.

[35] Cruchley S, Taylor MP, Ding R, Evans HE, Child DJ, Hardy MC (2015). Corrosion Science.

[36] Reynolds TD, Collins DM, Soor NK (2019). Acta Materialia.

[37] Wang M, Qu J, Yin T, Sheng J, Deng Q, Lu X (2010). Journal of Iron and Steel Research.

[38] Blacklocks AN, Atkinson A, Packer RJ, Savin SLP, Chadwick AV (2006). Solid State Ionics.

[39] Atkinson A, Levy MR, Roche S, Rudkin RA (2006). Solid State Ionics.

[40] Kuo Y-L, Hayashi S, Kakehi K (2021). Oxidation of Metals.

[41] Kawagishi K, Harada H, Sato A, Sato A, Kobayashi T (2006). The Journal of The Minerals, Metals & Materials Society.

[42] Song P, Liu M, Jiang X (2021). Materials & Design.

[43] Llewelyn SCH, Chater RJ, Jones NG, Hardy MC, Stone HJ (2021). Corrosion Science.

[44] Jacob KT, Saji VS, Reddy SNS (2007). The Journal of Chemical Thermodynamics.

[45] Crudden D, Mottura A, Warnken N, Raeisinia B, Reed R (2014). Acta Materialia.

[46] Pettit F, Meier G (1984). Superalloys.

[47] J. W. X. Wo, M. C. Hardy, and H. J. Stone, *Research Data Supporting “The Effect of Nb, Ta, and Ti on the Oxidation of a New Polycrystalline Ni-Based Superalloy*”, 10.17863/CAM.97038 10.1007/s11085-023-10218-7 PMC11078735 38736430

