# Supplementary material for: The Effect of Nb, Ta, and Ti on the Oxidation of a New Polycrystalline Ni-Based Superalloy
Source: High Temp Corros Mater. 2024 Jan 30;101(3):485–509. doi: 10.1007/s11085-023-10218-7 (PMC11078735; doi:10.1007/s11085-023-10218-7)
Supplement: Supplementary file 1 — Supplementary file1 (DOCX 15681 KB) [file 11085_2023_10218_MOESM1_ESM.docx]

# Supplementary Information for:

# The Effect of Nb, Ta, and Ti on the Oxidation of a New Polycrystalline Ni-Based Superalloy

J. W. X. Wo^1^, M. C. Hardy^2^ and H. J. Stone^1^*

^1^Department of Materials Science and Metallurgy, University of Cambridge, 27 Charles Babbage Road, Cambridge, CB3 0FS, UK

^2^Rolls-Royce plc, PO Box 31, Derby, DE24 8BJ, United Kingdom

*Corresponding author. Tel.: +44 (0)1223 334320, Email: [hjs1002@cam.ac.uk](mailto:hjs1002@cam.ac.uk)

**In journal: High Temperature Corrosion of Materials**


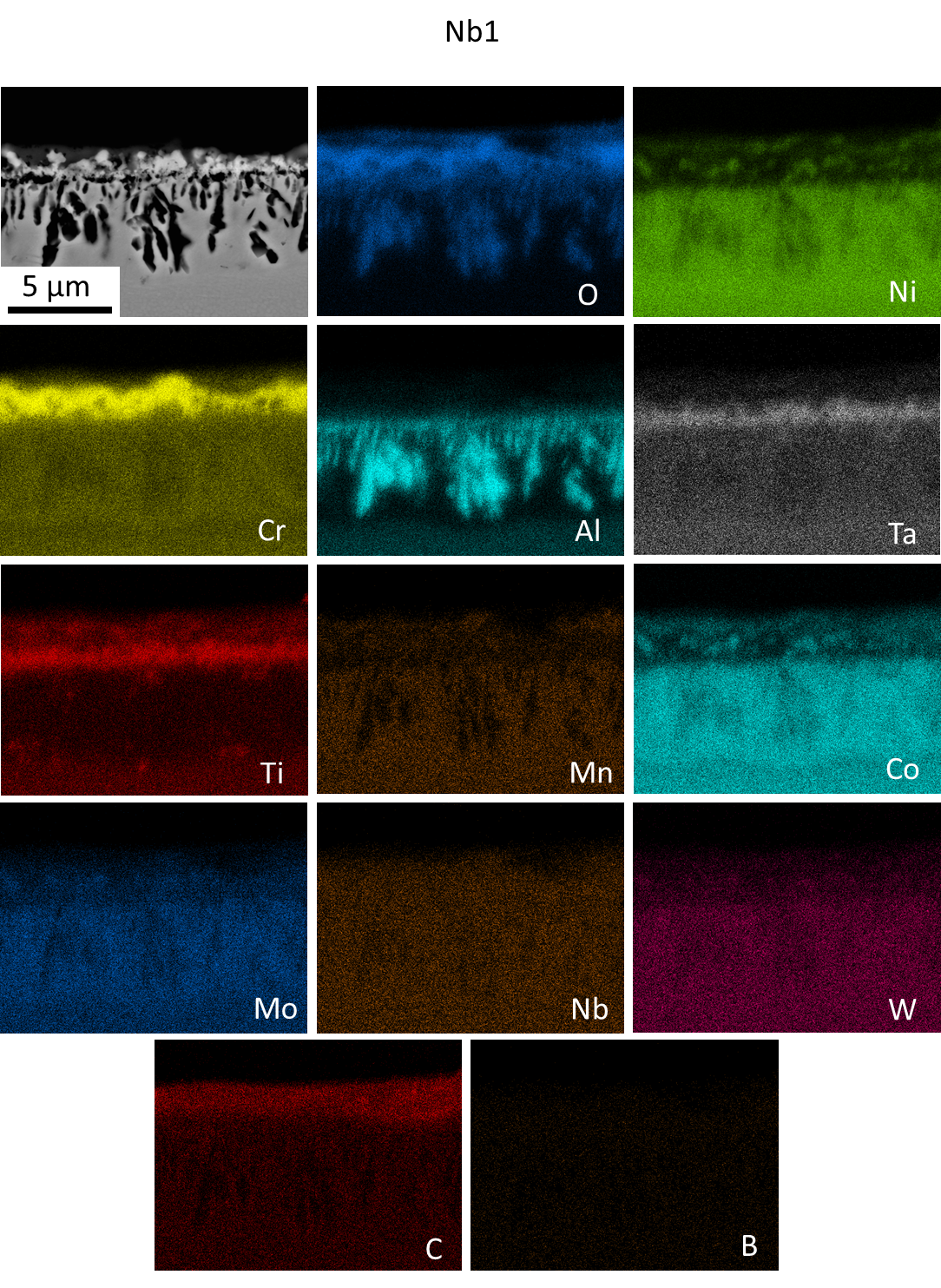


Figure 1 – BSE-SEM cross-sectional image and EDX elemental maps for Nb1 after oxidation at 800°C for 1000 hours.


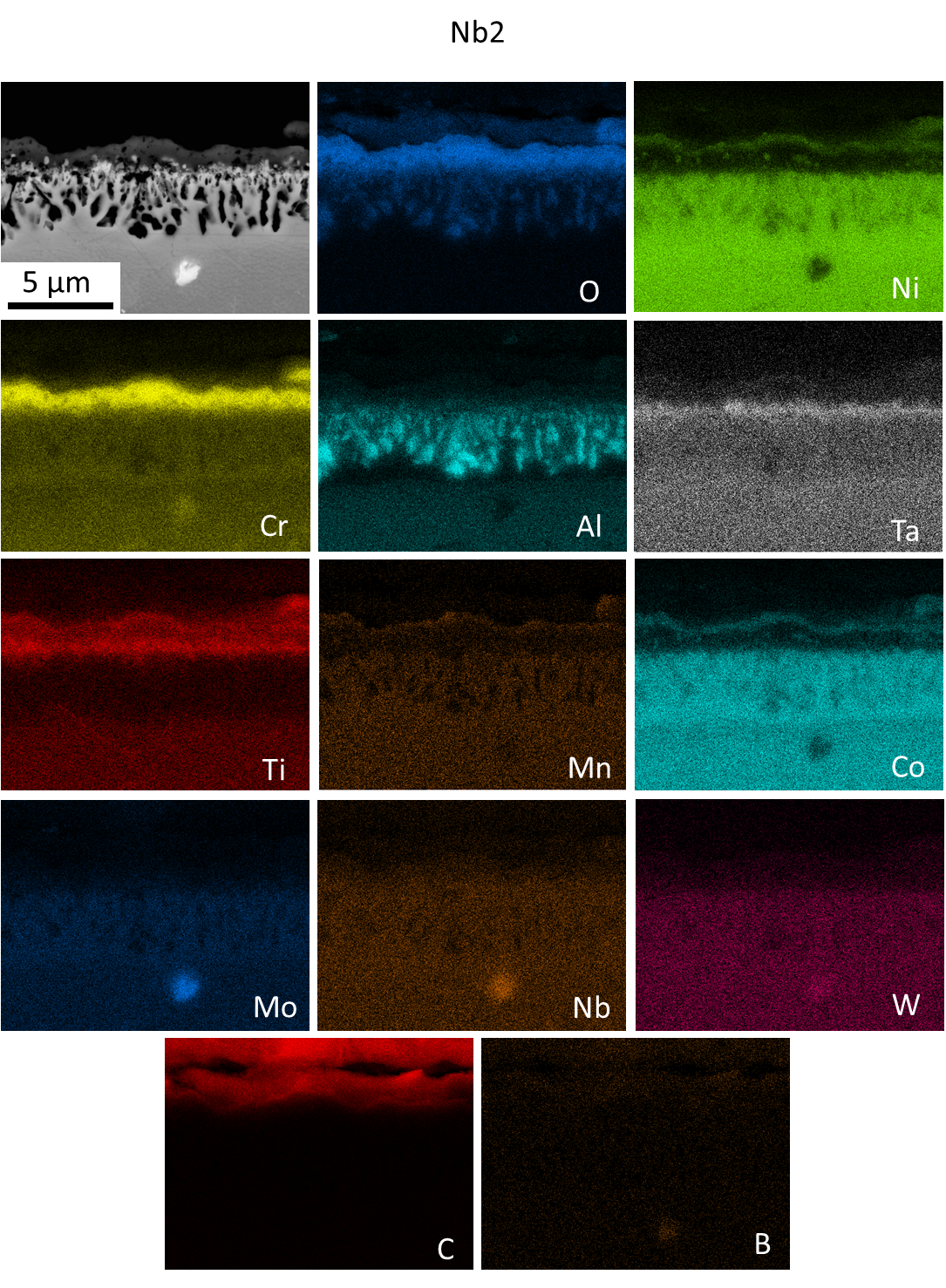


Figure 2 – BSE-SEM cross-sectional image and EDX elemental maps for Nb2 after oxidation at 800°C for 1000 hours.


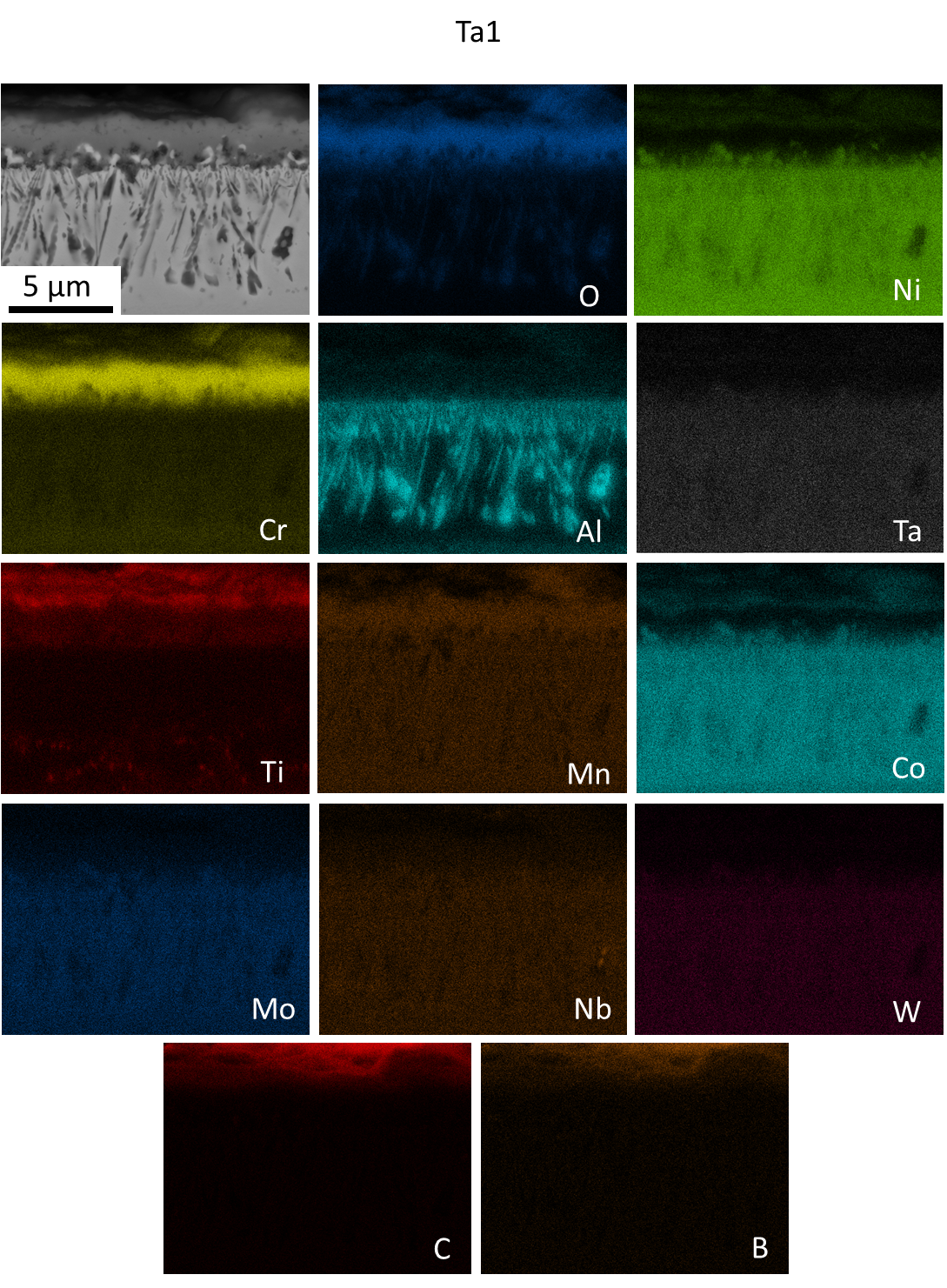


Figure 3 – BSE-SEM cross-sectional image and EDX elemental maps for Ta1 after oxidation at 800°C for 1000 hours.


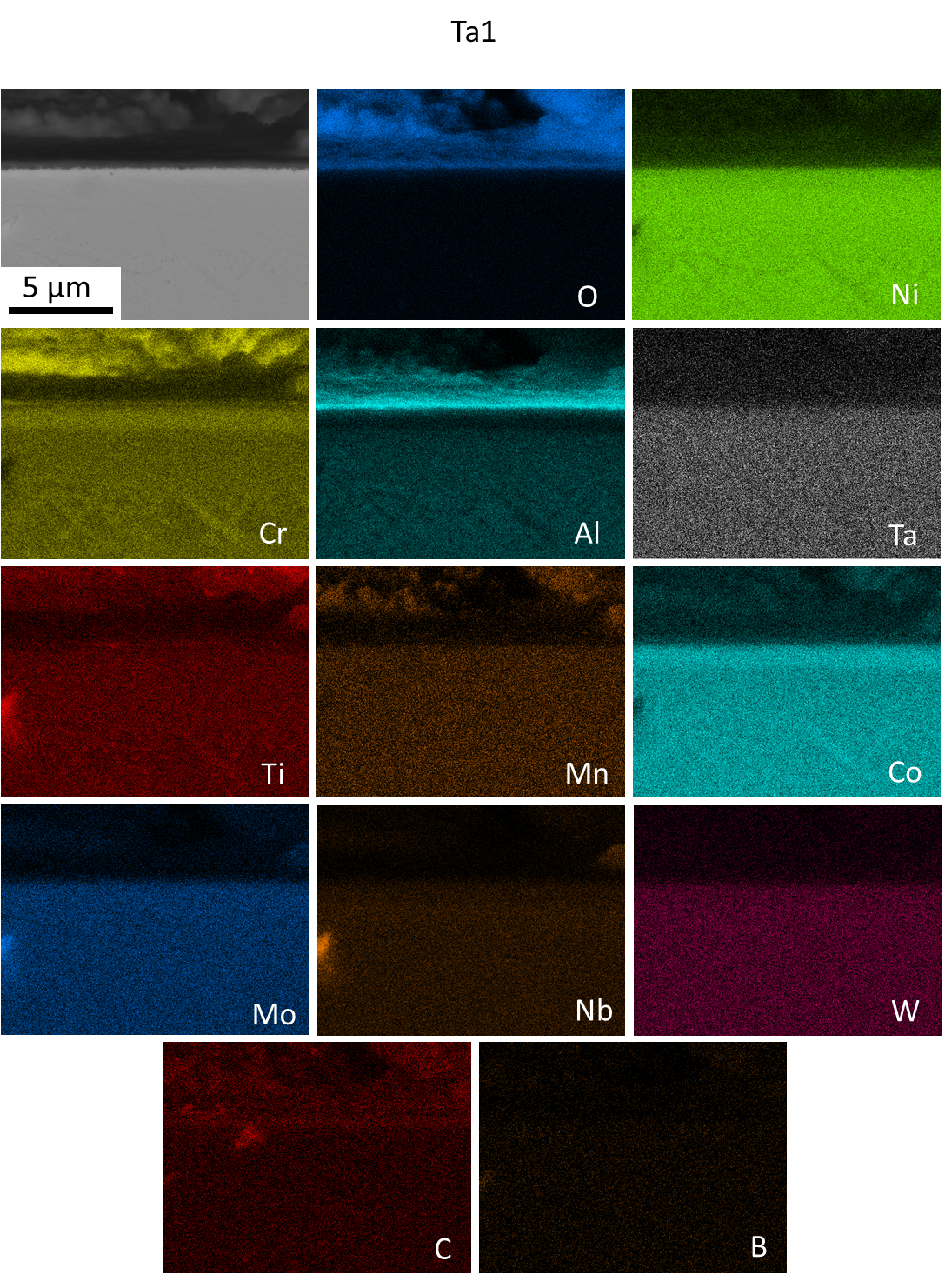


Figure 4 – BSE-SEM cross-sectional image and EDX elemental maps for an Al_2_O_3_-forming region in Ta1 after oxidation at 800°C for 1000 hours.


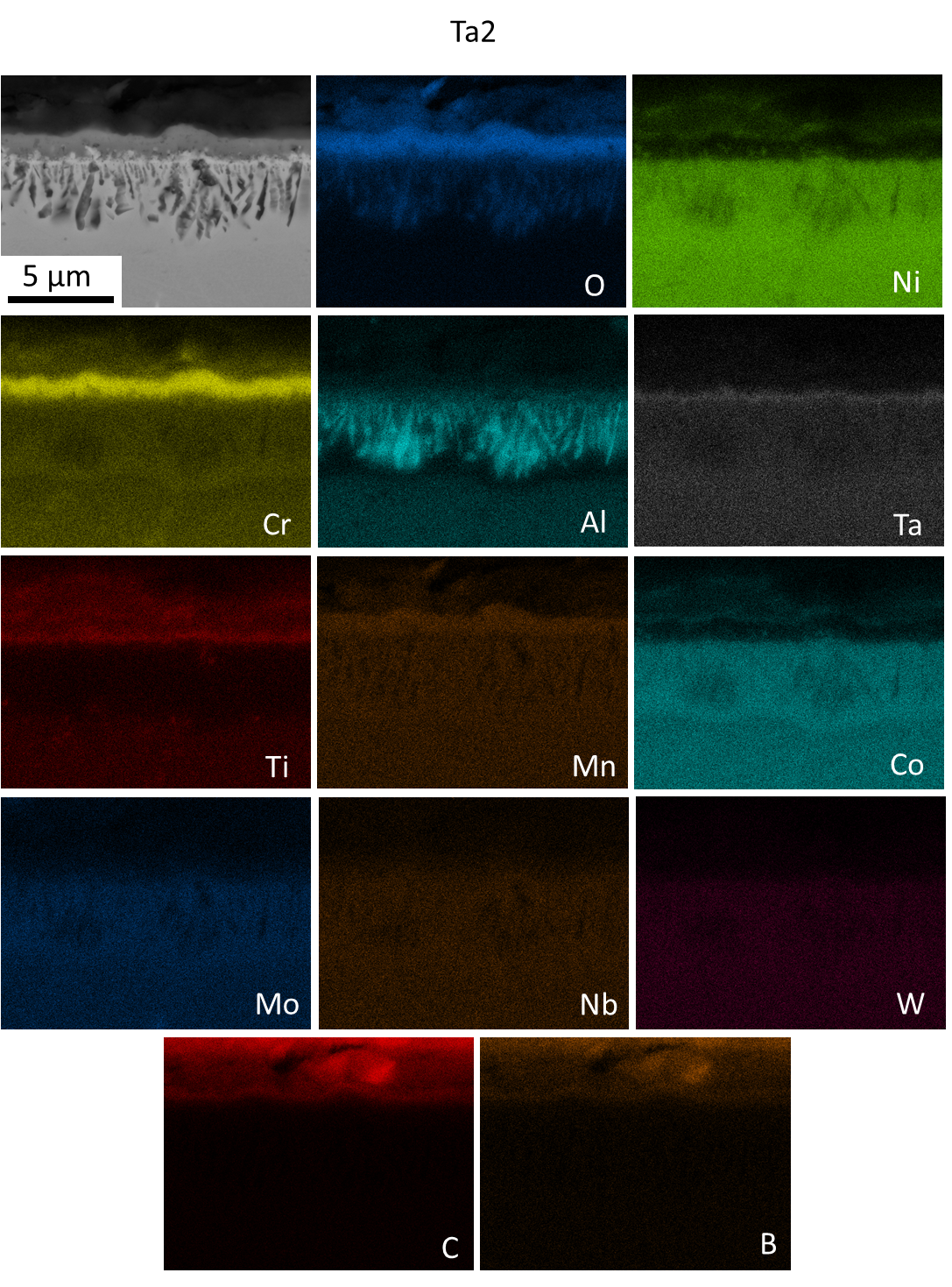


Figure 5 – BSE-SEM cross-sectional image and EDX elemental maps for Ta2 after oxidation at 800°C for 1000 hours.


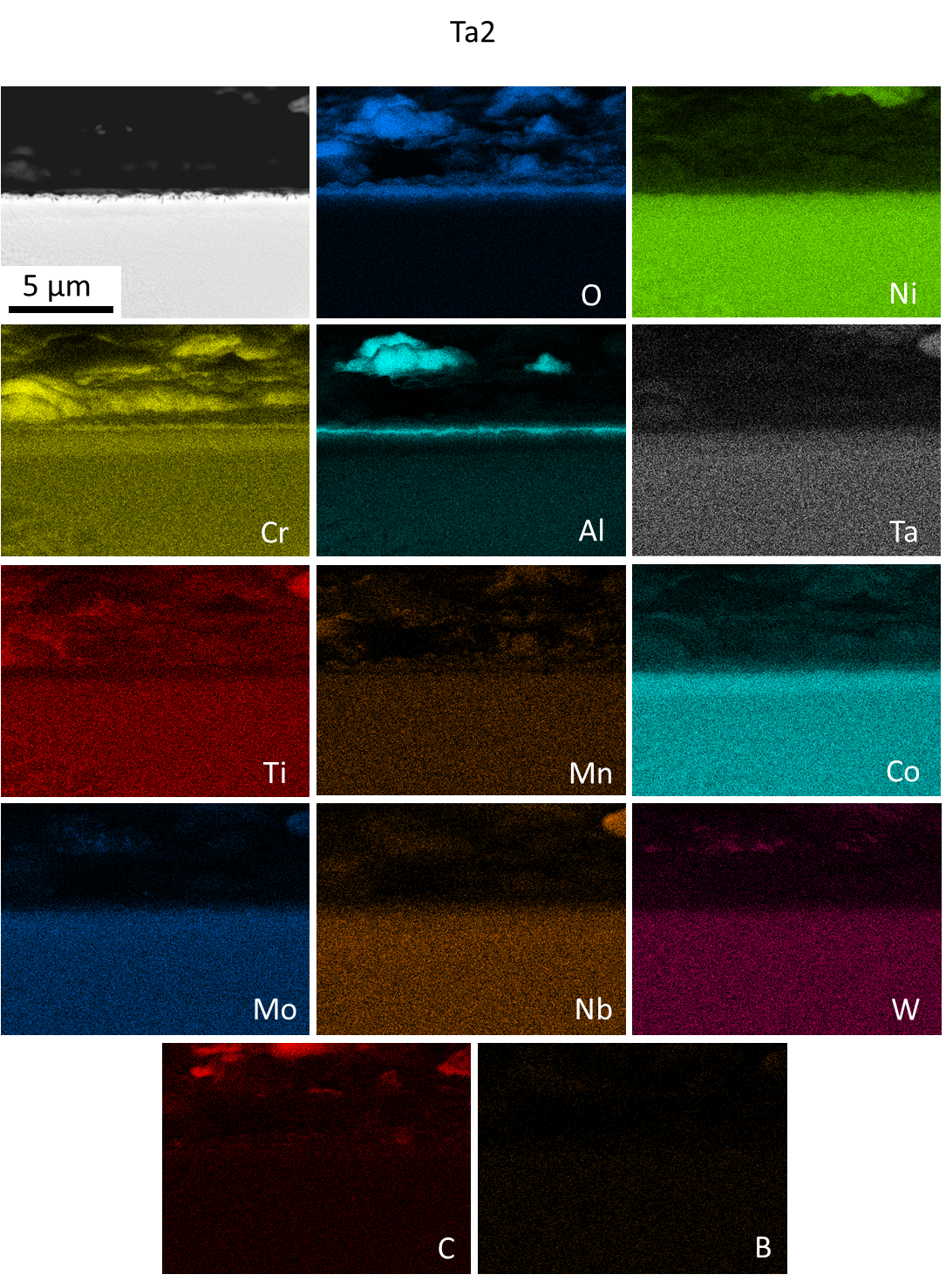


Figure 6 – BSE-SEM cross-sectional image and EDX elemental maps for an Al_2_O_3_-forming region in Ta2 after oxidation at 800°C for 1000 hours.


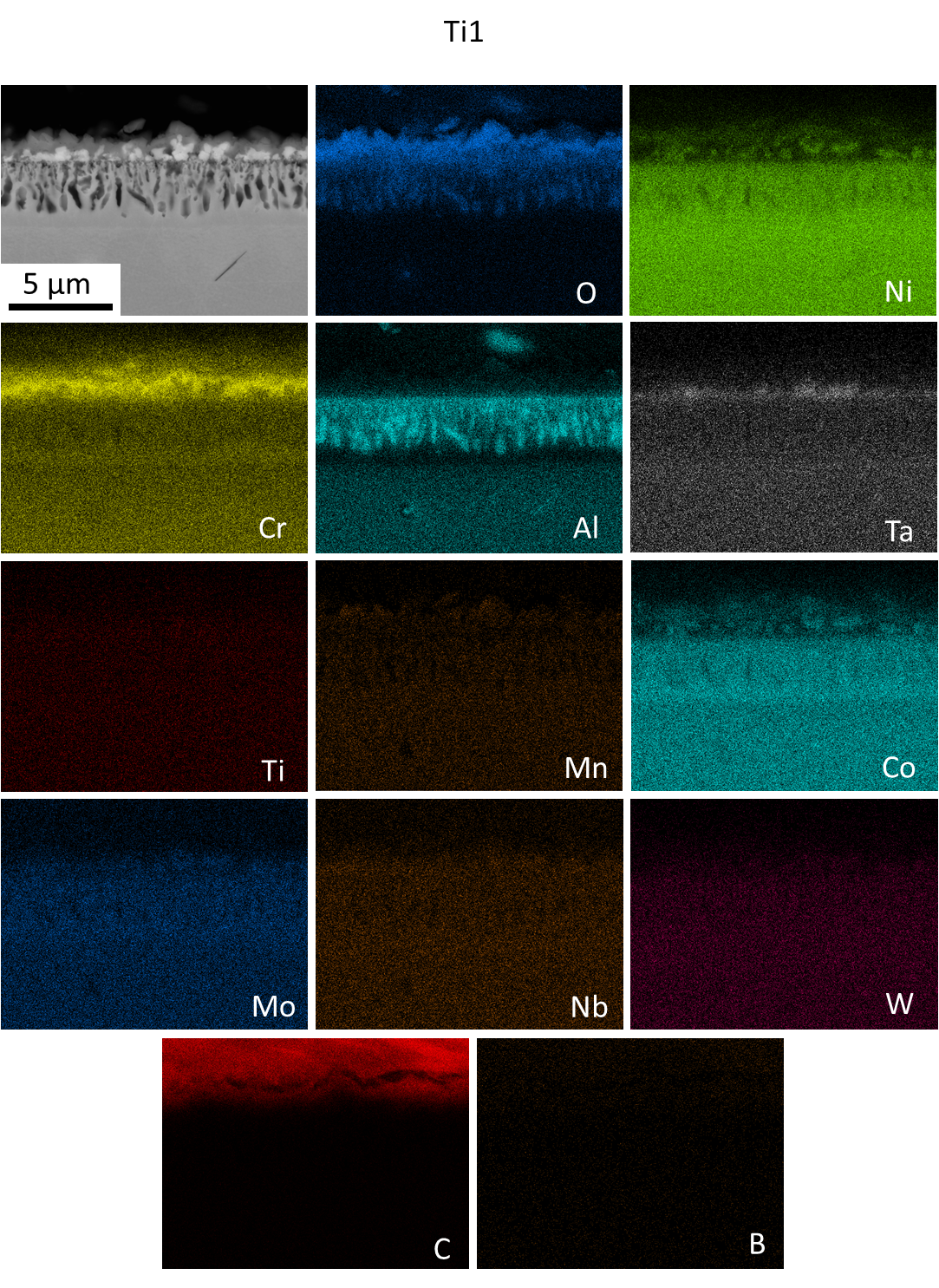


Figure 7 – BSE-SEM cross-sectional image and EDX elemental maps for Ti1 after oxidation at 800°C for 1000 hours.


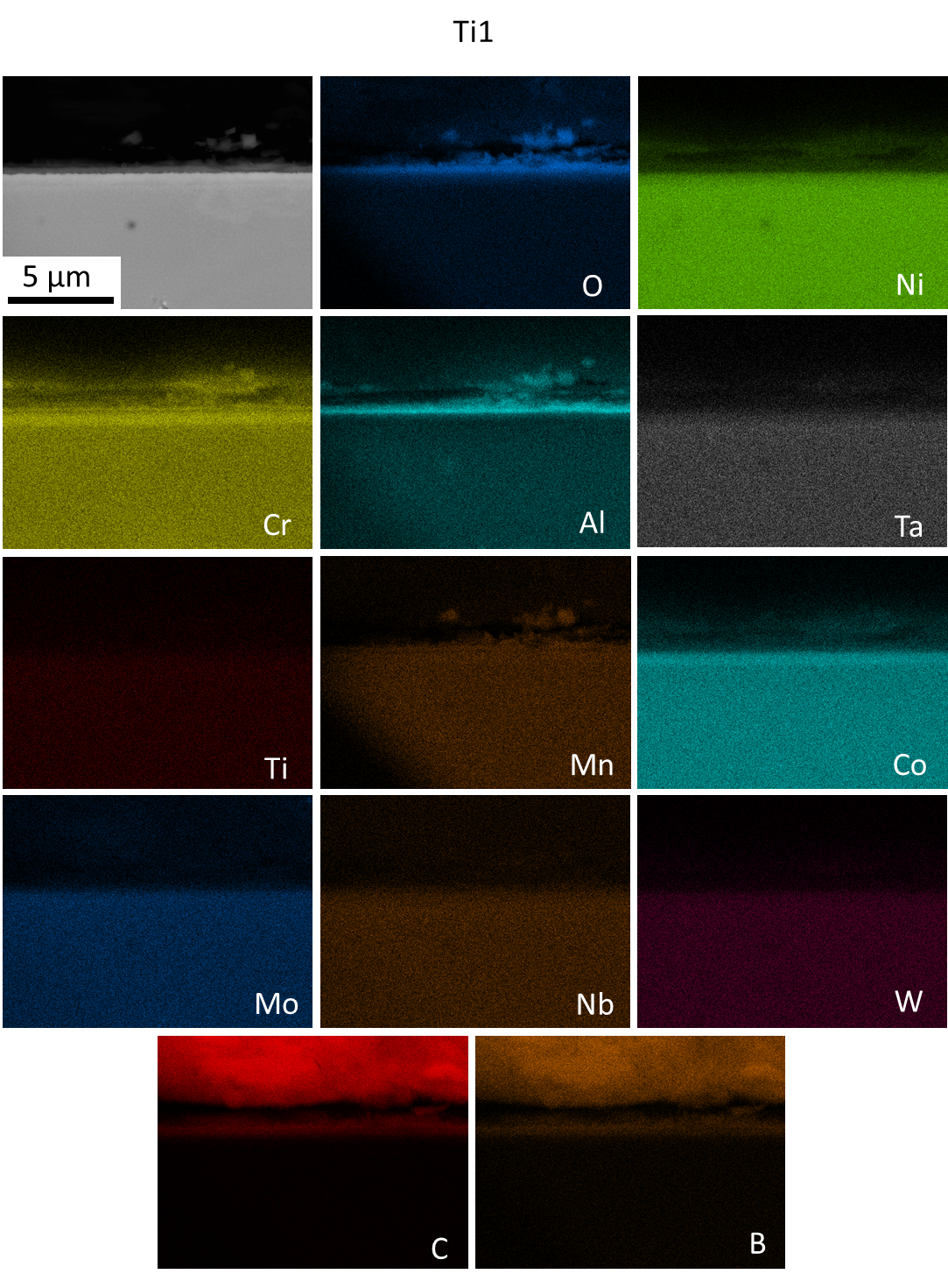


Figure 8 – BSE-SEM cross-sectional image and EDX elemental maps for an Al_2_O_3_-forming region in Ti1 after oxidation at 800°C for 1000 hours.


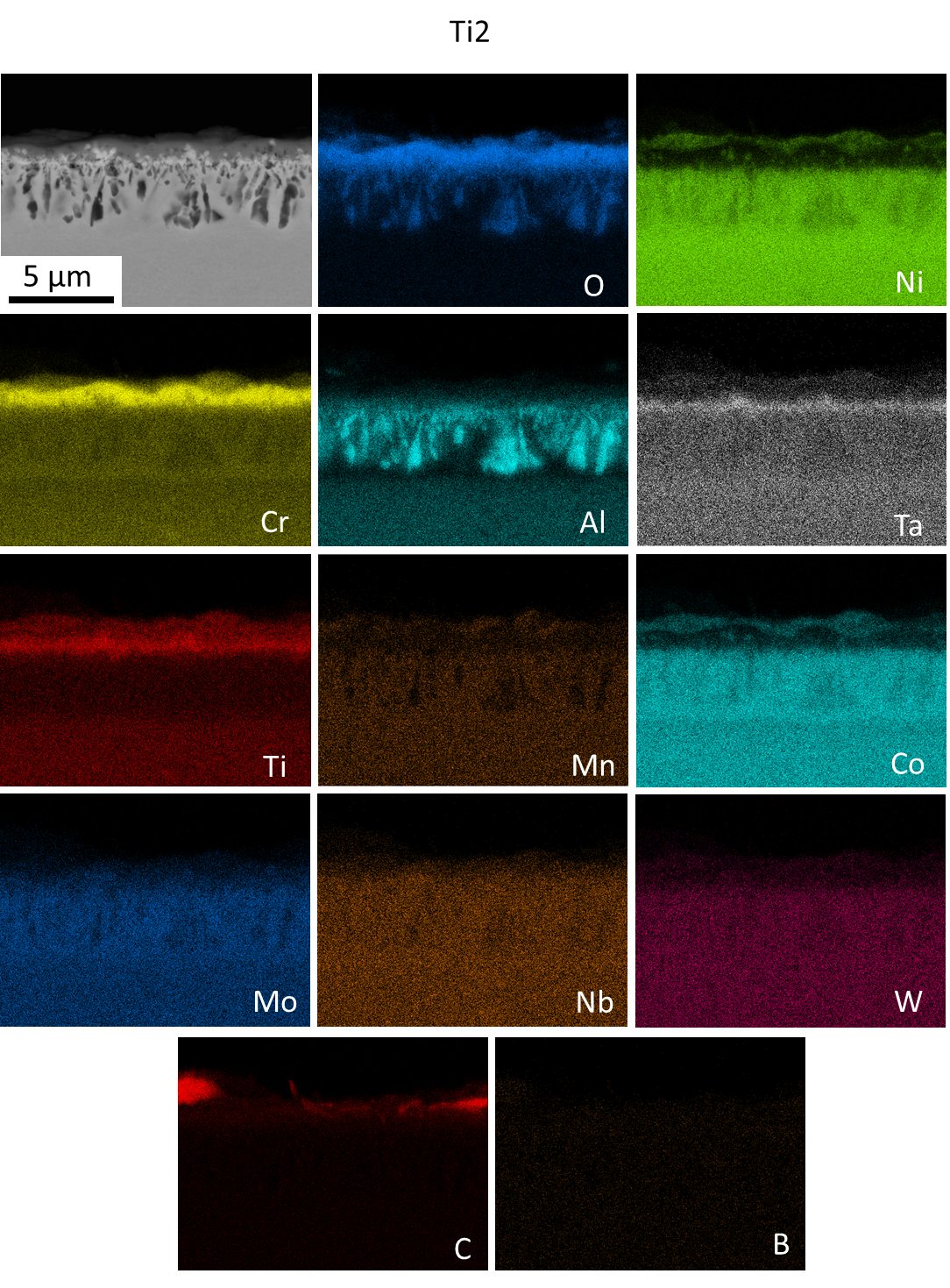


Figure 9 – BSE-SEM cross-sectional image and EDX elemental maps for Ti2 after oxidation at 800°C for 1000 hours.
